# OTUD1 deubiquitinase regulates NF-κB- and KEAP1-mediated inflammatory responses and reactive oxygen species-associated cell death pathways

**DOI:** 10.1038/s41419-022-05145-5

**Published:** 2022-08-08

**Authors:** Daisuke Oikawa, Min Gi, Hidetaka Kosako, Kouhei Shimizu, Hirotaka Takahashi, Masayuki Shiota, Shuhei Hosomi, Keidai Komakura, Hideki Wanibuchi, Daisuke Tsuruta, Tatsuya Sawasaki, Fuminori Tokunaga

**Affiliations:** 1 Department of Medical Biochemistry, Graduate School of Medicine, Osaka Metropolitan University, Osaka, Japan; 2 Department of Environmental Risk Assessment, Graduate School of Medicine, Osaka Metropolitan University, Osaka, Japan; 3 Division of Cell Signaling, Fujii Memorial Institute of Medical Sciences, Tokushima University, Tokushima, Japan; 4 Division of Cell-Free Sciences, Proteo-Science Center (PROS), Ehime University, Matsuyama, Japan; 5 Department of Molecular Biology of Medicine, Graduate School of Medicine, Osaka Metropolitan University, Osaka, Japan; 6 Department of Gastroenterology, Graduate School of Medicine, Osaka Metropolitan University, Osaka, Japan; 7 Department of Dermatology, Graduate School of Medicine, Osaka Metropolitan University, Osaka, Japan; 8 Department of Molecular Pathology, Graduate School of Medicine, Osaka Metropolitan University, Osaka, Japan

**Keywords:** Stress signalling, Cell death, Inflammation

## Abstract

Deubiquitinating enzymes (DUBs) regulate numerous cellular functions by removing ubiquitin modifications. We examined the effects of 88 human DUBs on linear ubiquitin chain assembly complex (LUBAC)-induced NF-κB activation, and identified OTUD1 as a potent suppressor. OTUD1 regulates the canonical NF-κB pathway by hydrolyzing K63-linked ubiquitin chains from NF-κB signaling factors, including LUBAC. OTUD1 negatively regulates the canonical NF-κB activation, apoptosis, and necroptosis, whereas OTUD1 upregulates the interferon (IFN) antiviral pathway. Mass spectrometric analysis showed that OTUD1 binds KEAP1, and the N-terminal intrinsically disordered region of OTUD1, which contains an ETGE motif, is indispensable for the KEAP1-binding. Indeed, OTUD1 is involved in the KEAP1-mediated antioxidant response and reactive oxygen species (ROS)-induced cell death, oxeiptosis. In *Otud1*^−/−^-mice, inflammation, oxidative damage, and cell death were enhanced in inflammatory bowel disease, acute hepatitis, and sepsis models. Thus, OTUD1 is a crucial regulator for the inflammatory, innate immune, and oxidative stress responses and ROS-associated cell death pathways.

## Introduction

The linear ubiquitin chain assembly complex (LUBAC), composed of HOIL-1L (also known as RBCK1), HOIP (RNF31), and SHARPIN, is an E3 complex that specifically generates the Met1(M1)-linked linear polyubiquitin chain [1, 2]. Upon stimulation by proinflammatory cytokines, such as TNF-α, LUBAC is recruited to the TNF receptor (TNFR), and conjugates M1-ubiquitin chains to NF-κB-essential modulator (NEMO) and receptor-interacting serine/threonine-protein kinase 1 (RIP1). The M1-ubiquitin chain functions as a scaffold to form the TNFR signaling complex I and recruit the canonical IκB kinase (IKK), composed of IKKα, IKKβ, and NEMO [3, 4]. The recruited IKK molecules are activated and leading to the NF-κB signaling. Moreover, the LUBAC is crucial for anti-apoptosis, and genetic defects and polymorphisms of LUBAC are associated with various disorders [2].

At present, the K11-, K63-, and M1-ubiquitin chains are known to be involved in TNFR signaling complex I [4], and deubiquitinating enzymes (DUBs), such as Cezanne (OTUD7B), OTULIN, CYLD, and A20, are reportedly involved in NF-κB regulation [5]. Cezanne [6] and OTULIN [7] selectively hydrolyze K11- and M1-ubiquitin chains, respectively. Moreover, OTULIN and CYLD bind to the N-terminal PUB domain of HOIP, which regulates LUBAC activity [8, 9]. In contrast, A20 does not cleave linear polyubiquitin chains, and instead, it binds to the M1-ubiquitin chain, resulting in NF-κB suppression [10]. However, the DUBs that physiologically regulate LUBAC functions have remained elusive.

In this study, we examined the effects of 88 human DUBs on LUBAC-mediated NF-κB activation, and identified OTUD1 (also known as DUBA7) [11], as the most potent down-regulator. We further identified that OTUD1 is involved not only in TNF-α-induced apoptosis and necroptosis but also in KEAP-1-mediated oxidative stress response and reactive oxygen species (ROS)-induced cell death, oxeiptosis.

## Materials and methods

A detailed method is available in the Online Supplemental Materials.

## Results

### OTUD1 is a negative regulator of canonical NF-κB signaling

**Fig. 1:**
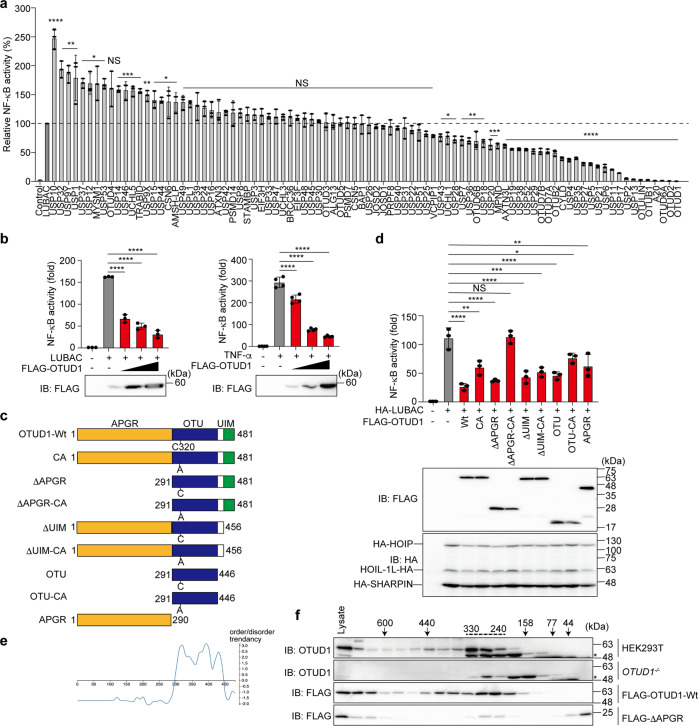
OTUD1 suppresses canonical NF-κB activation through the catalytic activity and the N-terminal region. **a** Screening for DUBs that regulate LUBAC-mediated NF-κB activation. Effects of 88 human DUBs on LUBAC-induced NF-κB activation were analyzed by a luciferase assay. **b** Dose-dependent inhibition by OTUD1 on LUBAC- and TNF-α-induced NF-κB activation. Effects of increasing amounts (0.1, 0.3, and 1.0 μg) of OTUD1 were examined with co-expression of LUBAC or 6 h treatment with 10 ng/ml TNF-α in HEK293T cells. **c** Domain structure of wild-type (Wt) and mutants of OTUD1. APGR: Ala-, Pro-, and Gly-rich region; OTU ovarian tumor protease, UIM ubiquitin-interacting motif. **d** Effect of OTUD1 mutants on the LUBAC-induced NF-κB activity. The relative NF-κB activity induced in the presence of Wt or various mutants of OTUD1, and expression levels of OTUD1 and LUBAC subunits are shown. **a**, **b**, **d** Data are shown as mean ± SD by ANOVA post-hoc Tukey test (*n* = 3 or 4). *P < 0.05, **P < 0.01, ***P < 0.001, ****P < 0.0001, NS not significant. **e** The N-terminal APGR region is disordered. Intrinsically ordered and disordered segments of OTUD1 were analyzed by DICHOT [12] (https://idp1.force.cs.is.nagoya-u.ac.jp/dichot/). **f** OTUD1 eluted in the high molecular weight fractions. Gel filtration analyses of lysates prepared from parental and *OTUD1*^*−/−*^ cells, and FLAG-OTUD1-Wt- and FLAG-ΔAPGR-expressing HEK293T cells were performed using a Superdex 200 column. Concentrated fractions were subjected to immunoblotting with the indicated antibodies. *Non-specific signal.

To comprehensively explore the DUBs involved in LUBAC-mediated NF-κB activation, we prepared 88 human DUB cDNAs and analyzed their effects by a luciferase assay in HEK293T cells (Fig. 1a). Taking the NF-κB activity induced by the expression of LUBAC alone as 100%, its co-expression with 16 DUBs, such as USP10, upregulated the NF-κB activity. In contrast, 34 DUBs significantly downregulated the LUBAC-induced NF-κB activation. Since OTUD6A, OTUD2, and OTUD1 had stronger inhibitory effects than those of the known LUBAC-suppressive DUBs, such as CYLD, OTULIN, and A20, we performed another NF-κB luciferase assay by expressing 10-fold higher amounts of LUBAC subunits than those in Fig. 1a. As a consequence, these DUBs dose-dependently suppressed the LUBAC- and/or TNF-α-induced NF-κB activation (Fig. 1b, Supplementary Fig. 1a, b). Since OTUD1 showed the most potent inhibitory effect, we focused on investigating its physiological functions.

Human OTUD1 is composed of an unknown N-terminal region (a.a. 1–290), an OTU domain (a.a. 291–446), which contains the active Cys320, and a ubiquitin-interacting motif (UIM, a.a. 457–481) (Fig. 1c) [11]. Although the N-terminal region lacks homology with known domains, it shares similarities with the amino acid sequence of the hypothetical protein Rv1157c of *Mycobacterium tuberculosis* (Supplementary Fig. 1c). Interestingly, the region contains an abundance of Ala (21.7%), Pro (16.9%), and Gly (9.6%). Therefore, we named the region as Ala-, Pro-, and Gly-rich region (APGR). To identify the critical region of OTUD1 in NF-κB inhibition, we constructed various mutants of OTUD1 and performed luciferase assays (Fig. 1c, d, Supplementary Fig. 1d). The OTUD1-Wt strongly suppressed the LUBAC- and TNF-α-induced NF-κB activities and the catalytically inactive OTUD1-CA showed partial NF-κB suppression. The combined mutation with the catalytically inactive APGR-deletion (ΔAPGR-CA) completely abolished the NF-κB-suppressive effect of OTUD1. These results suggested that both the catalytic activity and the APGR play a role in NF-κB suppression.

The protein disorder prediction by DICHOT [12] suggested that APGR is an intrinsically disordered low complexity domain (Fig. 1e). Moreover, the endogenous and transiently expressed FLAG-tagged OTUD1 (calculated molecular weight: 51 kDa) in HEK293T cells eluted in broad fractions in a gel filtration analysis, mainly at approximately 330–240 kDa (Fig. 1f). In contrast, FLAG-ΔAPGR (calculated molecular weight: 22 kDa) predominantly eluted at monomer fractions of <44 kDa. These results suggested that the APGR functions as a protein-interaction site.

### OTUD1 preferentially cleaves K63-linked ubiquitin chain

OTUD1 primarily cleaves K63-linked diubiquitin [11], but it also cleaves K6-, K11- and K48-chains [13, 14]. Furthermore, OTUD1 reportedly cleaves K33-chains in SMAD7 [15], and atypical K6-, K11-, and K29-chains from IRF3 [16]. Our biochemical analyses reconfirmed that the full-length FLAG-OTUD1-Wt in HEK293T cell lysates effectively converted K63-linked diubiquitin to monoubiquitin, among the 8 types of diubiquitins tested in vitro (Supplementary Fig. 1e). Moreover, recombinant GST-OTUD1 completely hydrolyzed the K63-linked polyubiquitin chain, and processed a long K48-linked polyubiquitin chain to tetraubiquitin (Supplementary Fig. 1f). However, OTUD1 did not cleave M1-chains, indicating that OTUD1 downregulates the NF-κB activation independently of the cleavage of linear ubiquitin chain, and principally by hydrolyzing the K63-linked ubiquitin chain.

### OTUD1 down-regulates inflammatory cytokine-induced canonical NF-κB activation

The *OTUD1* gene is composed of a single exon in both humans and mice, and we constructed *OTUD1*-knockout (*OTUD1*^*−/−*^) HeLa and HEK293T cells and *Otud1*^*−/−*^-mice (Supplementary Fig. 2a, b). The genetic depletion of *OTUD1* showed no effect on the expression of LUBAC and NF-κB signaling factors (Supplementary Fig. 2c). Moreover, OTUD1 is ubiquitously expressed in various mouse tissues (Supplementary Fig. 2d), and intracellular amounts of K48-linked, K63-linked, and pan-ubiquitin chains were not affected by the genetic ablation of *Otud1* in mouse embryonic fibroblasts (MEFs) (Supplementary Fig. 2e).

**Fig. 2:**
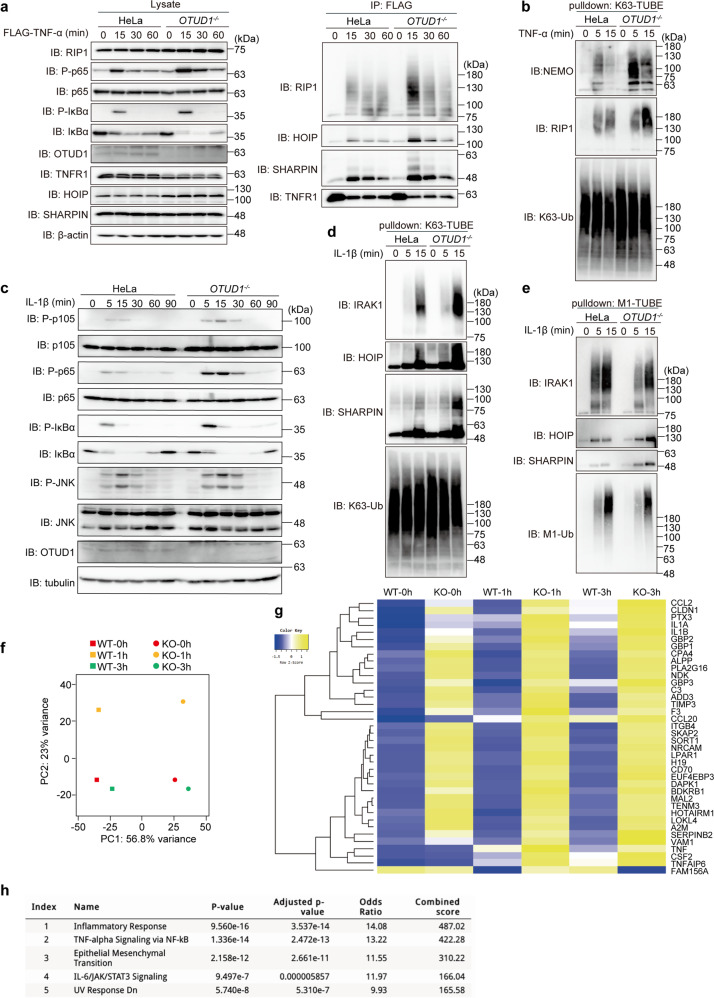
OTUD1 downregulates inflammatory cytokine-induced canonical NF-κB activation. **a** The enhanced ubiquitination and recruitment of NF-κB signaling factors to TNFR complex I. Parental and *OTUD1*^*−/−*^-HeLa cells were stimulated with 1 μg/ml FLAG-TNF-α for the indicated periods, and cell lysates and anti-FLAG immunoprecipitates were subjected to immunoblotting with the depicted antibodies. **b** K63-linked polyubiquitination of NF-κB activators is enhanced in *OTUD1*^*−/−*^ cells. Parental and *OTUD1*^*−/−*^-HeLa cells were stimulated with 20 ng/ml TNF-α for the indicated periods, and then pulled down by K63-TUBE. Samples were subjected to immunoblotting with the indicated antibodies. **c** The enhanced IL-1β-induced NF-κB activation in *OTUD1*^*−/−*^ cells. Parental and *OTUD1*^*−/−*^ cells were stimulated with 1 ng/ml IL-1β, and analyzed as in **a**. **d**, **e** Alterations of K63- and M1-linked ubiquitination of NF-κB activators in *OTUD1*^−/−^-HeLa cells. Parental and *OTUD1*^*−/−*^ cells were stimulated with 1 ng/ml IL-1β for the indicated periods, and pulled down by K63-TUBE (**d**) or M1-TUBE (**e**). Samples were immunoblotted with the indicated antibodies. **f**, **g** RNA-seq analysis. Parental and *OTUD1*^*−/−*^-HeLa cells were stimulated with 10 ng/ml IL-1β for 1 and 3 h. The cells were lysed and subjected to a transcriptome-wide expression analysis using their extracted total RNA. The principal component analysis (**f**) and heatmap analysis of significantly varied 34 mRNAs, using the cut-off value of 10 (**g**) was performed. **h** The enhanced inflammatory responses in *OTUD1*^*−/−*^-HeLa cells. Taking the cut-off value of 3.0, pathway analyses of 175 up-regulated genes were performed by MSigDB Hallmark 2020, and are listed in ascending order of *P*-value.

To examine the cellular functions of OTUD1, we analyzed the TNF-α-induced canonical NF-κB pathway. The genetic ablation of *OTUD1* in HEK293T cells enhanced the TNF-α-induced NF-κB luciferase activity (Supplementary Fig. 2f). In *OTUD1*^*−/−*^-HeLa cells, TNF-α-induced phosphorylation of IκBα and p65, hallmarks of NF-κB activation, was upregulated as compared to that in the parental cells (Fig. 2a). Moreover, the FLAG-TNF-α precipitation indicated that OTUD1 is recruited to TNFR upon TNF-α stimulation, and the polyubiquitinations of RIP1 and LUBAC in the TNFR signaling complex I were enhanced in *OTUD1*^*−/−*^-cells as compared to the parental cells (Fig. 2a, Supplementary Fig. 2g). TANK-binding kinase 1 (TBK1) and IKKε reportedly prevent TNF-induced cell death by RIP1 phosphorylation [17]. Importantly, the recruitment and phosphorylation of TBK1 and IKKε were reduced in the TNFR-signaling complex I from *OTUD1*^*−/−*^ cells (Supplementary Fig. 2h). Thus, OTUD1 reciprocally regulates TNF-α-mediated NF-κB activation and TBK1/IKKε-mediated signaling in HeLa cells. We further investigated the effect of the genetic ablation of *OTUD1* on TNF-α-induced K63-ubiquitination, by using a tandem ubiquitin-binding entity (TUBE) [18]. After TNF-α stimulation, the K63-polyubiquitinations of NEMO and RIP1 were enhanced in *OTUD1*^−*/*−^-HeLa cells (Fig. 2b), indicating that OTUD1 regulates K63-ubiquitination in complex I. Indeed, the expression of NF-κB target genes was enhanced in TNF-α-treated *OTUD1*^−*/*−^-HeLa cells (Supplementary Fig. 2i). These results clearly indicated that OTUD1 is a component of TNFR signaling complex I and regulates the K63-ubiquitination of NF-κB activators, such as RIP1, LUBAC, and NEMO, in the TNF-α signaling pathway.

Similarly, upon stimulation with IL-1β, the phosphorylation of NF-κB factors and the subsequent degradation of IκBα were enhanced in *OTUD1*^*−/−*^ cells as compared to the parental cells (Fig. 2c). In contrast, the phosphorylation of JNK, a MAP kinase, was not affected in *OTUD1*^*−/−*^-cells. When K63-polyubiquitinated proteins were captured from cell lysates by K63-TUBE, enhanced high molecular weight smear migrations of interleukin-1 receptor-associated kinase 1 (IRAK1), HOIP, and SHARPIN were detected in IL-1β-treated *OTUD1*^*−/−*^-HeLa cells (Fig. 2d). In contrast, the M1-TUBE analysis indicated that the M1-ubiquitination of these factors was not increased (Fig. 2e), suggesting that OTUD1 regulates the IL-1β-induced K63-deubiquitination of IRAK1 and LUBAC. After IL-1β-treatment, the mRNA and protein levels of NF-κB targets were enhanced in *OTUD1*^*−/−*^-cells, as compared to those in parental cells (Supplementary Fig. 2j, k).

To further investigate the effect of OTUD1 on signal transduction and transcription, we performed RNA-seq analysis. A principal component analysis clearly demonstrated that the *OTUD1*-deficiency affected the transcription of a set of genes (Fig. 2f). The gene ontology analyses revealed the significant upregulation of inflammatory responses and NF-κB-related factors in *OTUD1*^*−/−*^-HeLa cells (Fig. 2g, h, Supplementary Fig. 3a). These results clearly indicated that OTUD1 is a negative regulator for the IL-1β-induced canonical NF-κB activation pathway.

We further examined the role of OTUD1 using splenic B cells from Wt- and *Otud1*^*−/−*^-mice, and confirmed that the *Otud1*-deficiency did not affect the CD40- or B cell receptor-mediated induction of NF-κB target genes (Supplementary Fig. 3b, c). Furthermore, lymphotoxin β-mediated non-canonical NF-κB activation, which is demonstrated by the intranuclear translocation of p52 and RelB, was not affected in *Otud1*^*−/−*^-MEFs (Supplementary Fig. 3d).

### OTUD1 activates IFN antiviral signaling

**Fig. 3:**
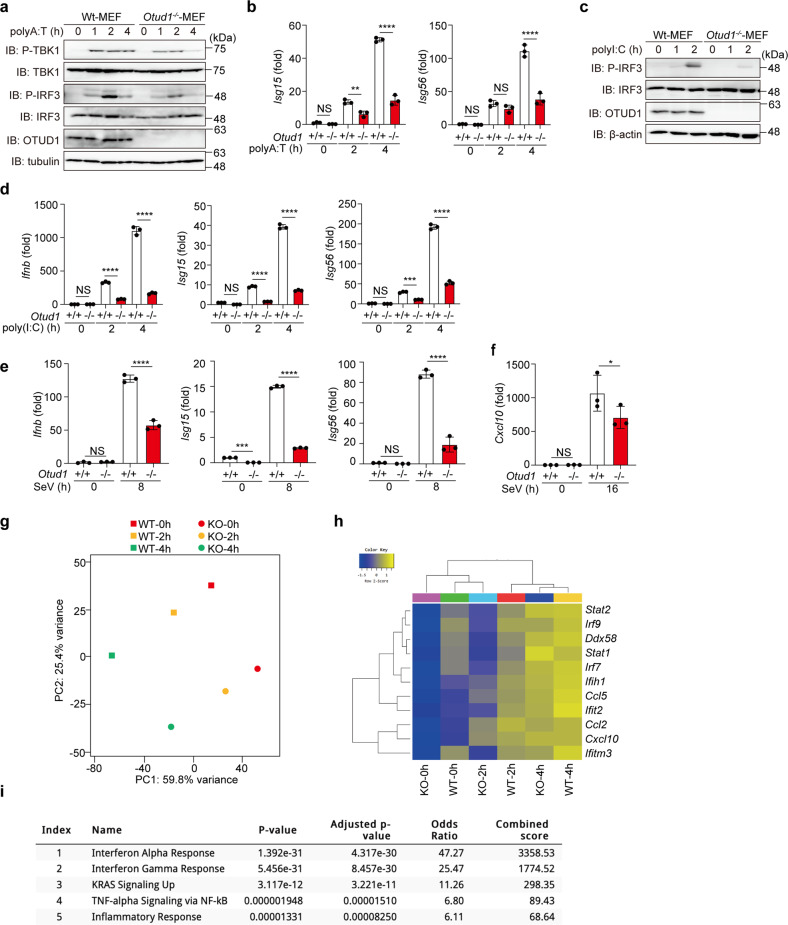
OTUD1 upregulates type I IFN antiviral pathway. **a** The poly(A:T)-mediated IFN antiviral pathway is suppressed in *Otud1*^*−/−*^-MEFs. *Otud1*^*+/+*^- and *Otud1*^*−/−*^-MEF cells were stimulated with 1 μg/ml poly(A:T) for the indicated periods, and the cell lysates were immunoblotted with the indicated antibodies. **b** The expression of IRF3-target genes is suppressed in poly(A:T)-treated *Otud1*^*−/−*^-MEF cells. MEFs were stimulated with 1 μg/ml poly(A:T) for the indicated periods, and the mRNA levels were assessed by qPCR. **c** The suppressed poly(I:C)-mediated antiviral response in *Otud1*^*−/−*^-MEFs. *Otud1*^*+/+-*^- and *Otud1*^*−/−*^-MEFs were stimulated with 10 μg/ml poly(I:C) for the indicated periods, and cell lysates were immunoblotted with the indicated antibodies. **d** Reduced poly(I:C)-mediated antiviral response in *Otud1*^*−/−*^-BMDMs. BMDMs from *Otud1*^*+/+-*^- and *Otud1*^*−/−*^-mice were stimulated with 10 μg/ml poly(I:C) for the indicated periods, and a qPCR analysis was performed. **e**, **f** Sendai virus (SeV)-induced antiviral response is suppressed in *Otud1*^*−/−*^-cells. *Otud1*^*+/+-*^- and *Otud1*^*−/−*^-MEFs (**e**) or BMDMs (**f**) were infected with SeV at a CIU of 10 for 1 h, incubated for the indicated periods, and then subjected to qPCR analyses. **b**, **d**–**f** Data are shown as mean ± SD by ANOVA post-hoc Tukey test (*n* = 3). *P < 0.05, **P < 0.01, ***P < 0.001, ****P < 0.0001, NS: not significant. **g–i** Suppressed transcription of IRF3-target genes in *Otud1*^*−/−*^-MEFs. *Otud1*^*+/+*^- and *Otud1*^*−/−*^-MEFs were stimulated with 10 μg/ml poly(I:C) for 2 and 4 h, and RNA-seq analyses were performed. **g** A principal component analysis of the RNA-seq analysis. **h** Taking the cutoff value of 10, significantly varied mRNAs are shown by the heatmaps. **i** Taking the cutoff value of 3.0, pathway analyses of 179 down-regulated genes were performed by MSigDB Hallmark 2020 and listed in ascending order of *P*-value.

We next examined the antiviral pathway using *Otud1*^*+/+*^- and *Otud1*^*−/−*^-MEFs. Upon stimulation with poly(dA:dT), which activates the IFN antiviral pathway [19], the phosphorylation of TBK1 and IRF3 and the expression of IRF3-target genes were reduced in *Otud1*^*−/−*^-MEF cells as compared to the *Otud1*^*+/+*^-MEFs (Fig. 3a, b). Similarly, the TLR3 ligand poly(I:C)-induced phosphorylation of IRF3 and the expression of IRF3-targets were decreased in *Otud1*^*−/−*^-bone marrow-derived macrophages (BMDMs) and MEFs (Fig. 3c, d, Supplementary Fig. 4a). Upon infection with Sendai virus (SeV), a single-stranded RNA virus, *Otud1*^*−/−*^-MEFs and -BMDMs showed reduced expression of IRF3-target genes as compared to *Otud1*^*+/+*^-cells (Fig. 3e, f). Indeed, RNA-seq analysis of poly(I:C)-treated MEFs revealed that the *Otud1*-deficiency downregulated the transcription of a set of IFN genes (Fig. 3g, h), and gene ontology analyses demonstrated that the *Otud1*-deficiency affected the expression of IFN and the RIG-I-like receptor signaling pathway (Fig. 3i, Supplementary Fig. 4b). Collectively, these results indicated that OTUD1 is a positive regulator for the type I IFN antiviral pathway.

### OTUD1 suppresses TNF-α-induced apoptosis and necroptosis

When TNFR signaling complex I fail to induce NF-κB activation, TNF-α stimulation induces apoptosis with the formation of complex II, which is composed of Fas-associated death domain protein (FADD) and caspase 8 [20, 21]. A pan-caspase inhibitor (ZVAD) suppresses apoptosis, whereas it induces another programmed necrosis, necroptosis, with the formation of necrosome by RIP1, RIP3, and then mixed lineage kinase domain-like pseudokinase (MLKL) [22].

**Fig. 4:**
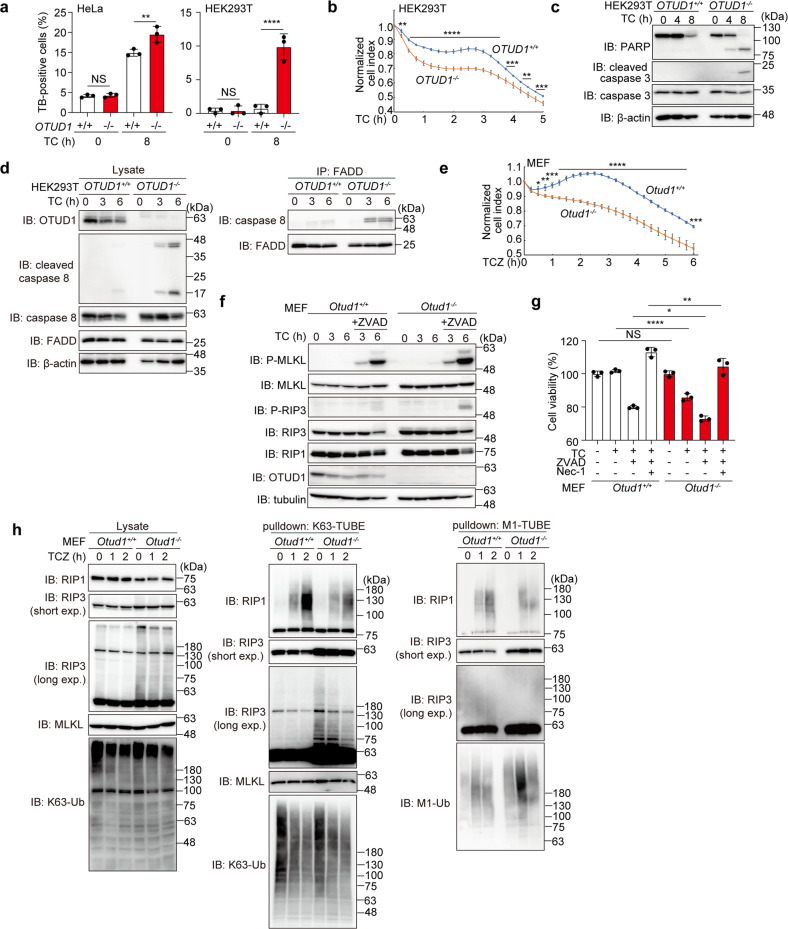
OTUD1 regulates TNF-α-induced apoptosis and necroptosis. **a** Increased TNF-α-induced cell death by the genetic ablation of *OTUD1*. Wt- and *OTUD1*^*−/−*^-HeLa and HEK293T cells were treated with or without 10 ng/ml TNF-α and 10 μg/ml CHX (TC), and trypan blue-positive cells were counted. **b** Reduced cell viability of *OTUD1*-deficient cells. Wt- and *OTUD1*^*−/−*^-HEK293T cells were treated with TC, and the cell proliferation was measured by xCELLigence real-time cell monitoring. **c** Enhanced apoptosis in *OTUD1*^*−/−*^-HEK293T cells. Wt- and *OTUD1*^*−/−*^-HEK293T cells were treated with TC, and cell lysates were immunoblotted with the indicated antibodies. **d** TNFR complex II formation was accelerated in *OTUD1*^*−/−*^-HEK293T cells. Wt- and *OTUD1*^*−/−*^-HEK293T cells were treated with TC as in (**c**). The cell lysates and anti-FADD immunoprecipitates were then blotted with the indicated antibodies. **e** Increased necroptosis in *Otud1*^*−/−*^-MEFs*. Otud1*^*+/+*^- and *Otud1*^*−/−*^-MEFs were treated with 10 ng/ml TNF-α, 10 μg/ml CHX, and 20 μM ZVAD (TCZ), and the cell proliferation were measured as in (**b**). **f** Increased phosphorylation of MLKL and RIP3 in TCZ-treated *Otud1*^*−/−*^-MEFs. MEFs were treated with TC with or without ZVAD as indicated, and the cell lysates were immunoblotted with the depicted antibodies. **g** Necrostatin-1 (Nec-1) rescued cell death. *Otud1*^*+/+*^- and *Otud1*^*−/−*^-MEFs were treated with TC, ZVAD, and/or 100 μM Nec-1 for 8 h, as indicated, and cell viability was analyzed by a Celltiter Glo assay. **h** Enhanced K63-ubiquitination of RIP3 in *Otud1*^*−/−*^-MEFs. MEFs were treated with TCZ for the indicated periods, and cell lysates and K63- or M1-TUBE precipitates were immunoblotted with the indicated antibodies. Data are shown as mean ± SD by ANOVA post-hoc Tukey test (**a**, **g**; *n* = 3) or *t*-test at each time point (**b**, **e**; *n* = 4). *P < 0.05, **P < 0.01, ***P < 0.001, ****P < 0.0001, NS not significant.

After an 8 h treatment with TNF-α + cycloheximide (TC), the trypan blue-positive dead cells were significantly increased in *OTUD1*^*−/−*^-HeLa and HEK293T cells (Fig. 4a). The cell survival analysis indicated that *OTUD1*^*−/−*^-HEK293T cells are more sensitive to cell death than the parental cells after TC-treatment (Fig. 4b). Indeed, the cleavages of PARP, caspase 3, and caspase 8, hallmarks of the apoptosis, were enhanced in TC-treated *OTUD1*^*−/−*^-HEK293T cells as compared to those in parental cells (Fig. 4c, d). The effective complex II formation, as shown by the enhanced association of caspase 8 with FADD, was detected in *OTUD1*^*−/−*^-HEK293T cells (Fig. 4d). These results suggested that OTUD1 has an inhibitory effect on the TNF-α-induced apoptosis.

We next investigated the involvement of OTUD1 in necroptosis using MEFs. In the presence of TC + ZVAD (TCZ), the *Otud1*^*−/−*^-MEFs died more rapidly than the *Otud1*^*+/+*^-MEFs (Fig. 4e). The phosphorylation of MLKL and RIP3 was enhanced in *Otud1*^*−/−*^-MEFs (Fig. 4f), and TCZ-induced cell death in *Otud1*^*−/−*^-MEFs was rescued by a RIP1 kinase inhibitor, necrostatin-1 [23] (Fig. 4g). Interestingly, the polyubiquitination of RIP3 was enhanced in *Otud1*^*−/−*^-MEFs (Fig. 4h, left panel), indicating that RIP3 is a possible endogenous substrate of OTUD1. When K63-ubiquitinated proteins were pulled down by K63-TUBE, the smeared migration of RIP1 was slightly suppressed upon stimulation with TCZ in *Otud1*^*−/−*^-MEFs (Fig. 4h, middle panel). In contrast, the M1-ubiquitination of RIP3 was not detected in either MEFs (Fig. 4h, right panel). These results indicated that OTUD1 is a critical regulator in TNF-α-mediated necroptosis pathways through the removal of K63-ubiquitin chains from RIP3 under steady-state conditions.

### OTUD1 regulates KEAP1-mediated oxidative stress response and cell death

**Fig. 5:**
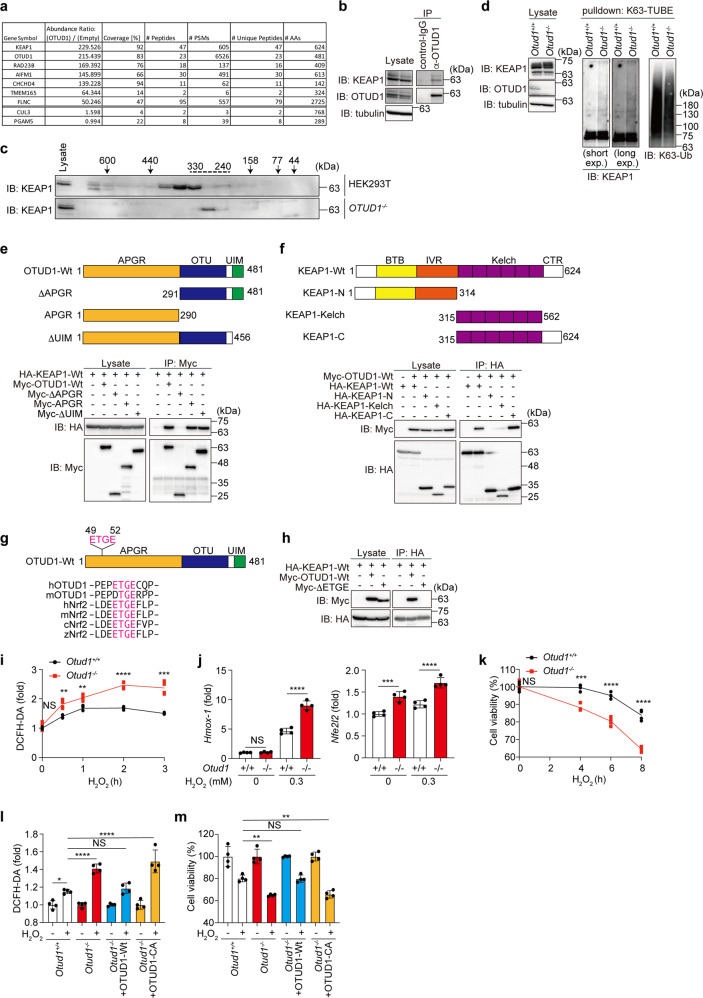
OTUD1 regulates KEAP1-mediated oxidative stress response and ROS-associated cell death. **a** Identification of OTUD1-interacting proteins. FLAG-tagged OTUD1 was expressed in HEK293T cells and immunoprecipitated with an anti-FLAG antibody, and the co-immunoprecipitants were then analyzed by MS. Proteins with an abundance ratio >50, and values for CUL3 and PGAM5 are shown. **b** OTUD1 physiologically interacts with KEAP1. HEK293T cell lysates and anti-OTUD1 immunoprecipitates were subjected to immunoblotting with the indicated antibodies. **c** KEAP1 in *OTUD1*^*−/−*^ cells eluted at a lower molecular weight as compared to that in parental cells. Gel filtration fractions from parental and *OTUD1*^*−/−*^ cells, as shown in Fig. 1f, were immunoblotted with an anti-KEAP1 antibody. **d** OTUD1 regulates K63- ubiquitination of KEAP1. Cell lysates and K63-TUBE precipitates from *Otud1*^+/+^- and *Otud1*^*−/−*^-MEFs were immunoblotted with the indicated antibodies. **e** APGR of OTUD1 is required for KEAP1-binding. Wt- and mutants of Myc-OTUD1 were expressed with HA-KEAP1 in HEK293T cells. Cell lysates and anti-Myc-immunoprecipitates were immunoblotted with the indicated antibodies. **f** The Kelch domain and the C-terminal region (CTR) of KEAP1 is responsible for OTUD1-binding. A similar analysis as in **e** was performed using Wt- and mutants of HA-KEAP1 and Myc-OTUD1. **g** OTUD1 contains an ETGE motif in APGR. Localization of the ETGE motif in OTUD1, and amino acid sequence alignment with NRF2 are shown [26]. h: human, m: mouse, c: chicken, z: zebrafish. **h** The ETGE motif in OTUD1 is indispensable for KEAP1-binding. Myc-tagged Wt- or ETGE motif-deleted mutant of OTUD1 was expressed with HA-KEAP1-Wt as indicated, and cell lysates and anti-HA immunoprecipitates were immunoblotted with the depicted antibodies. **i** Enhanced hydrogen peroxide-induced ROS generation in *Otud1*-deficient cells. *Otud1*^*+/+*^- and *Otud1*^*−/−*^-MEFs were treated with 0.3 mM H_2_O_2_ for the indicated periods, and the intracellular ROS levels were analyzed by a DCFH-DA assay. **j** Enhanced expression of NRF2 target genes in *Otud1*^*−/−*^-MEFs. MEFs were treated with or without 0.3 mM H_2_O_2_ for 3 h, and qPCR analyses were performed. **k** Reduced cell viability in *Otud1*^*−/−*^-MEFs under oxidative stress. MEFs were treated with 0.3 mM H_2_O_2_ for the indicated periods, and cell viability was analyzed by Celltiter Glo assay. **l** DUB activity of OTUD1 is necessary to suppress ROS production. OTUD1-Wt or active site mutant (CA) was restored into *Otud1*^*−/−*^-MEFs, and ROS levels were analyzed by a DCFH-DA assay after treatment with or without 0.3 mM H_2_O_2_ for 2 h. **m** DUB activity of OTUD1 protects cells from H_2_O_2_-induced death. A similar treatment as in **l** was performed with or without 0.1 mM H_2_O_2_ for 8 h, and cell viability was analyzed by a Celltiter Glo assay. Data are shown as mean ± SD by *t*-test of each time point (*n* = 4, **i**, **k**) or ANOVA post-hoc Tukey test (*n* = 4, **j**, **l**, **m**). *P < 0.05, **P < 0.01, ***P < 0.001, ****P < 0.0001, NS not significant.

To further examine the physiological roles of OTUD1, proteins interacting with FLAG-OTUD1 in HEK293T cells were analyzed by immunoprecipitation-mass spectrometry. Then, we identified KEAP1 as the most prominent interactor of OTUD1 (Fig. 5a, Supplementary Fig. 5a). KEAP1 regulates the proteasomal degradation of the NRF2 transcription factor, as a component of the Cullin 3 (CUL3)–E3 complex [24]. Moreover, KEAP1 is involved in excess reactive oxygen species (ROS)-induced cell death, named oxeiptosis, with the apoptosis-inducing factor mitochondria associated 1 (AIFM1) and the mitochondrial Ser/Thr protein phosphatase (PGAM5) [25]. Indeed, peptides derived from AIFM1, CUL3, and PGAM5 were identified in FLAG-OTUD1 co-precipitates (Fig. 5a, Supplementary Fig. 5a). We detected the endogenous association of KEAP1 and OTUD1 (Fig. 5b). Gel filtration followed by an immunoblotting analysis showed that KEAP1 eluted in the ~410–330 kDa fractions in HEK293T cells (Fig. 5c), and as shown in Fig. 1f, OTUD1 co-eluted with KEAP1 in the 330 kDa fraction. In contrast, KEAP1 eluted in the ~280 kDa fraction in *OTUD1*^*−/−*^ cells. These results clearly indicated that the OTUD1-KEAP1 interaction exists under physiological conditions. Interestingly, the K63-polyubiquitination of KEAP1 was enhanced in *Otud1*^*−/−*^-MEFs (Fig. 5d). We further confirmed the K63-polyubiquitination of KEAP1 in *OTUD1*^*−/−*^-HEK293T cells by using an HA-tagged only-K63-ubiquitin mutant and a K63-ubiquitin-specific antibody (Supplementary Fig. 5b, 5c). Co-expression of OTUD1-Wt, but not OTUD1-CA, suppressed the K63-ubiquitination of KEAP1, suggesting that OTUD1 targets KEAP1 that removes the K63-ubiquitin chain. Moreover, we determined that the APGR of OTUD1 interacts with the C-terminal Kelch and CTR of KEAP1 (Fig. 5e, f). Importantly, we found that the human and mouse OTUD1 proteins contain ETGE and a similar DTGE sequence, respectively, in APGR, which plays the KEAP1-binding motif in NRF2 [26] (Fig. 5g). Indeed, the deletion of the ETGE motif from OTUD1 abolished the KEAP1-binding ability (Fig. 5h). Upon H_2_O_2_-treatment, ROS production was upregulated in *Otud1*^*−/−*^-MEFs as compared to *Otud1*^*+/+*^-MEFs (Fig. 5i), resulting in the enhanced expression of antioxidant genes at the early phase (~3 h) (Fig. 5j). However, prolonged treatment with H_2_O_2_ caused drastic cell death in *Otud1*^*−/−*^-MEFs, suggesting that the excessive ROS production subsequently induces oxeiptosis (Fig. 5k).

Interestingly, the restoration of OTUD1-Wt, but not the catalytically inactive mutant, rescued ROS resistance and cell viability (Fig. 5l, m), suggesting that the DUB activity of OTUD1 is necessary to resist oxeiptosis. OTUD1 directly bound with KEAP1, and PGAM5 co-precipitated with OTUD1 only in the presence of KEAP1 (Supplementary Fig. 5d). Although the co-precipitation of endogenous AIFM1 with FLAG-OTUD1 was detected by the MS analysis (Fig. 5a, Supplementary Fig. 5a), the co-precipitation of exogenous OTUD1 with AIFM1 was not detected (Supplementary Fig. 5d). Whereas, in *Otud1*^*−/−*^-MEFs, increased intranuclear AIFM1 was detected concomitantly with the reduced mitochondrial AIFM1 (Supplementary Fig. 5e), which may affect mitochondrial oxidative phosphorylation and ROS generation [27]. These results indicated that OTUD1 binds and regulates the K63-ubiquitination of KEAP1 under basal conditions and is important for the efficient antioxidative stress response, ROS production, and oxeiptosis. The increased H_2_O_2_-mediated ROS-production and reduced cell viability in *Otud1*^*−/−*^-MEFs than that in *Otud1*^*+/+*^-MEFs were prevented by broad antioxidant scavengers, such as *N*-acetylcysteine (NAC) and butylated hydroxyanisole (BHA), but not by a mitochondria-targeted superoxide dismutase mimetic, Mito-TEMP (Supplementary Fig. 5f, g). These results suggested that cytosolic ROS scavengers ameliorate the increased ROS levels and cell death in *Otud1*-deficient cells.

In addition to H_2_O_2_ treatment, the ROS levels were also upregulated in *Otud1*^*−/−*^-MEFs upon apoptotic (TC) and necroptotic (TCZ) stimuli, although TNF-α alone showed no effects on ROS production (Supplementary Fig. 5h). Since the enhanced ROS production was canceled by the restoration of Otud1-Wt, but not -CA, in TC- or TCZ-treated *Otud1*^*−/−*^-MEFs, the catalytic activity of OTUD1 is necessary to regulate TNF-α-induced cell death pathways (Supplementary Fig. 5i). Collectively, these results indicated that OTUD1 activity regulates TNF-α-induced apoptosis and necroptosis, as well as KEAP1-mediated oxeiptosis.

### OTUD1 suppresses inflammatory and oxidative stress responses in vivo

**Fig. 6:**
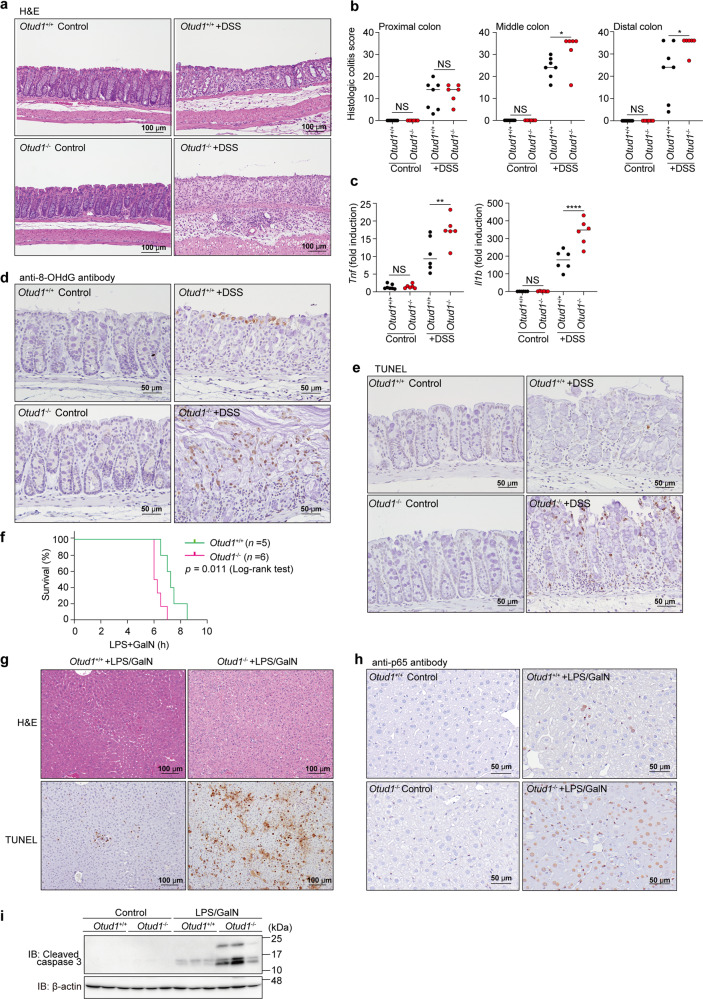
Enhanced inflammation, oxidative damage, and cell death in mouse inflammatory disease models. **a**, **b** Colon damage in DSS-administered *Otud1*^*−/−*^-mice. *Otud1*^*+/+*^- and *Otud1*^*−/−*^-mice were treated with 2.5% DSS for 7 days and then sacrificed. Representative images of H&E staining of distal colon (**a**) and histologic scores of colitis **b** (*n* = 6–7) are indicated. *Bars*; 100 µm. **c** Increased expression of NF-κB target genes in DSS-treated *Otud1*^*−/−*^-mice. qPCR analysis of NF-κB targets in the distal colon from DSS-treated mice (*n* = 6). Data are shown as scatter plots and evaluated by Mann–Whitney test. *P < 0.05, **P < 0.01, ****P < 0.0001, NS: not significant. **d**, **e** Increased oxidative DNA damage and cell death in DSS-administered *Otud1*^*−/−*^-mice. Specimens as in **a** were stained for 8-OHdG (**d**) or TUNEL (**e**). *Bars*: 50 µm. **f** *Otud1*^*−/−*^-mic**e** were labile for the LPS/GalN-induced acute hepatitis model. After injections of LPS (10 μg/kg) and GalN (800 mg/kg), the % survivals of *Otud1*^*+/+*^-(*n* = 5) and *Otud1*^*−/−*^-(*n* = 6) mice were determined by the Kaplan–Meier method with the Log-rank test. **g** Enhanced liver damage in *Otud1*^*−/−*^-mice. H&E and TUNEL staining of livers from *Otud1*^*+/+*^- and *Otud1*^*−/−*^-mice after 5 h administration of LPS/GalN. *Bars*: 100 µm. **h** Increased intranuclear localization of p65 in hepatocytes from LPS/GalN-treated *Otud1*^*−/−*^-mice. The specimens in **g** were stained with an anti-p65 antibody. *Bars*: 50 µm. **i** Increased caspase 3 activations in hepatocytes from *Otud1*^*−/−*^-mice. After a 5 h treatment with LPS/GalN, livers were excised, lysed, and immunoblotted with the indicated antibodies.

A recent report showed that OTUD1 inhibits colonic inflammation in vivo [28]. We confirmed that significant shortening of the colon, and higher diarrhea and fecal blood scores were observed in a DSS-administrated ulcerative colitis-like inflammatory bowel disease (IBD) model of *Otud1*^*−/−*^-mice (Supplementary Fig. 6a, b). The middle and distal colon regions in DSS-treated *Otud1*^*−/−*^-mice showed more severe damage, characterized by multiple focal dropouts of entire crypts, inflammatory cell infiltration, and edema, as compared with the DSS-treated *Otud1*^*+/+*^ mice (Fig. 6a). Histologic scores of colitis were significantly higher in the middle and distal colon of DSS-treated *Otud1*^*−/−*^-mice, as compared with DSS-treated *Otud1*^*+/+*^ mice (Fig. 6b). Moreover, the expressions of NF-κB target genes were upregulated in the distal colon from *Otud1*^*−/−*^-mice (Fig. 6c). Importantly, we detected the increased 8-OHdG-positive oxidative DNA damage and TUNEL-positive cell death in the colon of DSS-treated *Otud1*^*−/−*^-mice (Fig. 6d, e). Furthermore, p65 staining was predominantly observed in the cytoplasm of the colonic epithelial cells and the nuclei of a few inflammatory cells (Supplementary Fig. 6c, *Arrows*) in the lamina propria of the colonic mucosa in the control mice. Nuclear p65-positive colonic epithelial cells (Supplementary Fig. 6c, *Arrowheads*) were observed in the remaining colonic mucosa of DSS-treated *Otud1*^*+/+*^- and *Otud1*^*−/−*^-mice. Moreover, increased numbers of nuclear p65-positive colonic epithelial cells and inflammatory cells in the DSS-treated *Otud1*^*−/−*^-mice were consistent with the severity of colitis. These findings indicated that the canonical NF-κB activation is increased in DSS-induced colitis in the *Otud1*^*−/−*^-mice, as compared with the *Otud1*^*+/+*^-mice. In contrast, we did not detect any drastic effects on K63-, K48- and pan-ubiquitination in the colonic lysates from *Otud1*^*+/+*^- and *Otud1*^*−/−*^-mice, with or without DSS-treatment (Supplementary Fig. 6d). DSS-treated *Otud1*^*−/−*^-mice also showed splenomegaly with increased TUNEL-positive splenocytes (Supplementary Fig. 6e, f). These results indicated that OTUD1 suppresses the NF-κB-mediated inflammatory and ROS-induced oxidative damage responses, as well as cell death through specific deubiquitination in an in vivo colitis model.

To further clarify the pathophysiological role of OTUD1 in vivo, we adopted LPS/GalN-induced acute hepatitis and LPS-induced sepsis models, which are associated with inflammatory and oxidative responses [29–31]. The LPS/GalN-treated *Otud1*^*−/−*^-mice showed significantly shorter survival and remarkably increased hepatocellular death with an enhanced necroptosis score (Fig. 6f, g, Supplementary Fig. 7a, b). NF-κB p65 was negative (score 0) in hepatocytes from *Otud1*^*+/+*^- and *Otud1*^*−/−*^-mice. In contrast, positive p65 staining was observed in the nuclei of the hepatocytes in the LPS/GalN-treated *Otud1*^*+/+*^- (score 1) and *Otud1*^*−/−*^- (score 2) mice (Fig. 6h, Supplementary Fig. 7c). The intranuclear localization of p65 was significantly increased in the hepatocytes of the LPS/GalN-treated *Otud1*^*−/−*^-mice in comparison with the LPS/GalN-treated *Otud1*^*+/+*^-mice, as evidenced by the findings that the canonical NF-κB activation was significantly increased in the LPS/GalN-treated *Otud1*^*−/−*^-mice. Moreover, caspase 3 activation was upregulated in the LPS/GalN-treated *Otud1*^*−/−*^-mice (Fig. 6i). These results indicated that OTUD1 protects against acute hepatitis through the suppression of NF-κB activation and cell death in vivo.

In contrast to LPS/GalN, LPS challenge alone induces systemic inflammation and sepsis with increases in oxidative stress and NF-κB activation [32, 33]. When mice were intraperitoneally administered LPS, we found that the survival of *Otud1*^*−/−*^-mice was significantly shorter as compared to that of *Otud1*^*+/+*^-mice (Supplementary Fig. 7d). Histopathological findings in the livers are shown in Supplementary Table 1 and Supplementary Fig. 7e. In male mice, mild fatty changes characterized by increased microvacuoles in the cytoplasms of hepatocytes were observed in all LPS-treated *Otud1*^*+/+*^- and *Otud1*^*−/−*^-mice, but not in the *Otud1*^*+/+*^ and *Otud1*^−/−^ controls (Supplementary Fig. 7e, upper panels). In the female mice, the incidence of hepatocellular death (necrosis and/or apoptosis), predominantly localized in the midzonal area, was increased in the LPS-treated *Otud1*^*−/−*^-mice as compared with the LPS-treated *Otud1*^*+/+*^-mice, albeit without statistical significance (Supplementary Table 1, Supplementary Fig. 7e, lower panels). This suggested that the female *Otud1*^*−/−*^-mice exhibited higher susceptibility to LPS-induced hepatotoxicity than their wild-type counterparts. Indeed, the AST activities were significantly elevated in LPS-treated *Otud1*^*−/−*^-mice, as compared to the LPS-treated *Otud1*^*+/+*^-mice (Supplementary Fig. 7f). These results indicated that the genetic ablation of *Otud1* enhances septic shock.

### OTUD1 is associated with prognosis of kidney cancer

**Fig. 7:**
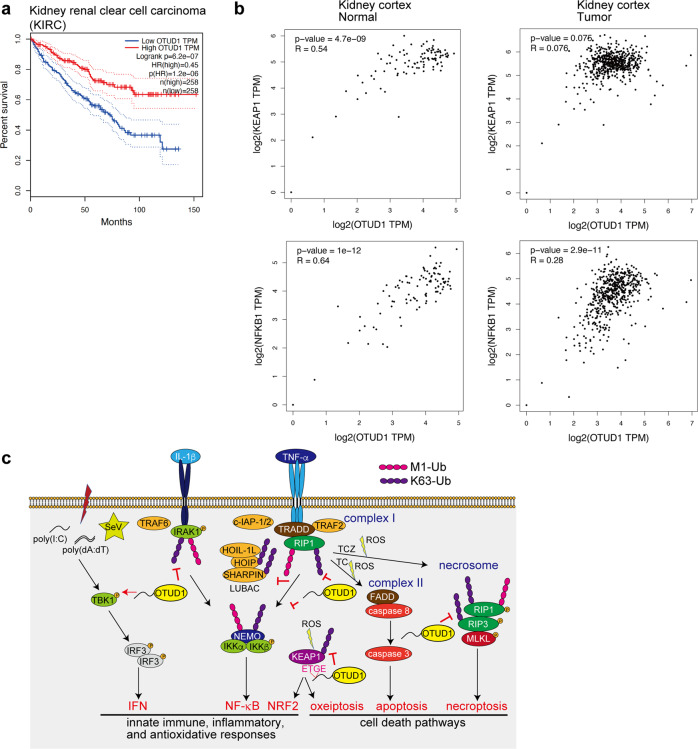
OTUD1 suppresses kidney cancer. **a** Low expression of *OTUD1* is a poor prognosis factor in kidney cancer. Kaplan–Meier survival curves of patients with kidney renal clear cell carcinoma (KIRC) with high (*n* = 258 patients) and low (*n* = 258 patients) expression of *OTUD1* mRNA, determined by the Log-rank test, are shown. **b** Loss of positive correlation between *OTUD1* and *KEAP1* in kidney cancer. Correlations of expression levels with Pearson correlations and *P*-values of *OTUD1* vs*. KEAP1* (*upper panels*) or *OTUD1* vs*. NF-κB1* (*lower panels*) in normal (*left panels*) and tumor (*right panels*) kidney cortex are shown. **c** Schematic representation of OTUD1 functions in the regulation of inflammatory and KEAP1-mediated oxidative stress responses, and cell death pathways.

Finally, to investigate the involvement of OTUD1 in human diseases, we analyzed cancer databases. We found that low levels of *OTUD1* expression are significantly associated with a poor prognosis in renal clear cell carcinoma patients (Fig. 7a). Importantly, the expressions of OTUD1 and KEAP1 were significantly correlated in the normal kidney cortex; however, the positive correlation was lost in the tumor (Fig. 7b). In contrast, the expression levels of OTUD1 and NF-κB1 were correlated in both the normal and tumor kidney cortexes. Thus, we concluded that the orchestration of OTUD1 with KEAP1 functions to suppress kidney cancer in humans, and therefore, OTUD1 is a critical regulator to maintain homeostasis.

## Discussion

OTUD1 was initially found as a biomarker of thyroid cancer [34] that reportedly stabilizes the p53 tumor suppressor [35]. OTUD1 also upregulates the expression of p21 and Mdm2, thus accelerating apoptosis [35], and cleaves K33-linked polyubiquitin chains from the TGF-β pathway inhibitor SMAD7 [15]. Furthermore, OTUD1 is induced by RNA viruses, which gives rise to the upregulation of Smurf1 [36]. OTUD1 reportedly regulates the Hippo pathway [37]. Furthermore, quite recently OTUD1 was found to suppress IBD by removing the K63 ubiquitin chain from RIP1 [28]. Collectively, these findings indicate that OTUD1 functions as a crucial regulator of cancer progression, antiviral host defense responses, and inflammatory responses.

We characterized the APGR as a disordered region (Fig. 1). APGR is required to suppress canonical NF-κB activation, exhibits a broad elution profile in gel filtration, and possesses a KEAP1-interaction motif (Figs. 1 and 5). Therefore, APGR is indispensable for the physiological function of OTUD1. We further found that OTUD1 is a component of TNFR complex I, and the K63-polyubiquitinations of various canonical NF-κB-signaling factors, such as IRAK1, LUBAC subunits (HOIP and SHARPIN), NEMO, and RIP1, were increased upon inflammatory cytokine-stimulation in *OTUD1*-knockout cells, suggesting that these proteins are potential substrates of OTUD1 (Supplementary Fig. 2 and Figs. 2, 7c).

Although the TNF-α-induced NF-κB activation was upregulated in *OTUD1*^*−/−*^-cells, the activation of TBK1 and IKKε were suppressed (Supplementary Fig. 2), and in contrast to the previous report [16], we determined that IRF3-mediated IFN antiviral signaling was down-regulated by the deficiency of *Otud1* (Fig. 3). Since TBK1 activation and IRF3 phosphorylation were suppressed in *Otud1*^*−/−*^-MEFs, OTUD1 seems to be primarily involved in the upstream TBK1/IKKε activation in the type I IFN production pathway (Fig. 7c).

DUBs such as CYLD [38], A20 [39], and OTUB1 [40] are reportedly involved in the regulation of the K63-ubiquitination of RIP1/RIP3. In this study, we showed that the genetic deficiency of *OTUD1* accelerated the TNF-α-induced apoptosis and necroptosis, and the basal K63 ubiquitination of RIP3 was enhanced in *Otud1*^*−/−*^-MEFs (Figs. 4, 7c), suggesting that RIP3 is an endogenous substrate of OTUD1 under physiological conditions.

Importantly, we identified OTUD1 as a physiological interactor with KEAP1 through the ETGE motif in APGR (Figs. 5, 7c). KEAP1 senses ROS and protects against oxidative stress as a component of the E3 complex to induce the proteasomal degradation of NRF2 [24]. The DLG and ETGE motifs in the Neh2 domain of NRF2 are crucial for KEAP1-binding [26], and the C-terminal Kelch domain of KEAP1 binds NRF2 as well as OTUD1. We showed that the K63-ubiquitination of basal KEAP1 was upregulated in *Otud1*^*−/−*^-MEFs, indicating that OTUD1 is a physiological regulator of KEAP1. At present, several E3s, such as TRAF6 [41], TRIM25 [42], CKIP1, and SMURF1 [43], reportedly ubiquitinate NRF2-KEAP1. Therefore, OTUD1 may antagonize these E3s.

In the presence of excess amounts of ROS, KEAP1 induces cell death named oxeiptosis through binding with PGAM5 and AIFM1 [25]. Moreover, upon TNF-α-induced necroptosis, PGAM5 binds necrosomes, composed of RIP1–RIP3–MLKL, which mediate the activation of the mitochondrial fission factor Drp1 and mitochondrial fragmentation [44]. A recent report found that OTUD1 is involved in caspase-dependent and -independent apoptosis by facilitating the intranuclear translocation of AIFM1 and the degradation of MCL1 [27]. We showed that OTUD1 is a regulator of the KEAP1–PGAM5–AIFM1 axis, which affects ROS generation and cell death pathways upon oxidative and inflammatory responses (Figs. 5, 7c and Supplementary Fig. 5).

NF-κB and NRF2/KEAP1 are redox-sensitive transcription factors that cooperate in oxidative stress and inflammatory responses and cell death pathways [45]. The intracellular levels of ROS, such as hydrogen peroxide, are regulated by the activities of thioredoxin reductase 1 and glutathione disulfide reductase [46, 47], and OTUD1 seems to be an additional crosstalk regulator of the NF-κB and NRF2/KEAP1 pathways. Furthermore, TNF-α reportedly induces ROS production through the activation of NADPH oxidase 1 complex (Nox1) [48], and RIP1 is necessary for ROS generation at the initiation of caspase-independent cell death [49, 50]. In this study, we showed the drastic increase in ROS levels by the TC- and TCZ-treatments, which accompanied the marked cell death in *Otud1*^*−/−*^-MEFs (Fig. 7c, Supplementary Fig. 5). Free radicals induce oxidative damage, which causes genotoxicity, oxidations, and cell death. Together, KEAP1-NRF2 and NF-κB co-operate the oxidative stress and inflammatory responses, and dysfunctions of these systems are associated with various disorders, such as neurodegeneration and inflammatory diseases [51, 52]. This study has demonstrated that OTUD1 is a central regulatory factor in these signal pathways, and the genetic ablation of *OTUD1* is associated with inflammatory bowel disease, hepatitis, sepsis, and kidney cancer (Figs. 6, 7 and Supplementary Figs. 6, 7) [16, 28]. Therefore, OTUD1 is a critical drug discovery target for the treatment of these diseases.

## Supplementary information

Supplementary Information

checklist

## Data Availability

The datasets generated during and/or analyzed during the current study are available from the corresponding author on reasonable request.
