# Supplementary material for: OTUD1 deubiquitinase regulates NF-κB- and KEAP1-mediated inflammatory responses and reactive oxygen species-associated cell death pathways
Source: Cell Death Dis. 2022 Aug 8;13(8):694. doi: 10.1038/s41419-022-05145-5 (PMC9360000; doi:10.1038/s41419-022-05145-5)
Supplement: Supplementary file 1 — Supplementary Information [file 41419_2022_5145_MOESM1_ESM.pdf]

ONLINE SUPPLEMENTARY MATERIALS

OTUD1 deubiquitinase regulates NF-κB- and KEAP1-mediated inflammatory responses and reactive oxygen species-associated cell death pathways

Daisuke Oikawa, Min Gi, Hidetaka Kosako, Kouhei Shimizu, Hirotaka Takahashi, Masayuki Shiota, Shuhei Hosomi, Keidai Komakura, Hideki Wanibuchi, Daisuke Tsuruta, Tatsuya Sawasaki and Fuminori Tokunaga

MATERIALS AND METHODS

Reagents and plasmids

The following reagents were obtained as indicated: human TNF-α and IL-1β (BioLegend), poly(dA:dT) and high molecular weight poly(I:C) (Invivogen), LPS from *E. coli* O111:B4 (Invivogen), cycloheximide (Sigma-Aldrich), recombinant mouse CD40 ligand (R&D Systems), α-IgM (Southern Biotech), zVAD-FMK (ZVAD, Peptide Institute, Inc.), *N*-acetylcysteine (NAC, Wako-Fujifilm), Mito-TEMP (MedChemExpress), butylated hydroxyanisole (BHA, Santa Cruz), and necrostatin-1 (ENZO Life Sciences). The open reading frames of cDNAs, including 88 human DUBs, were amplified by reverse transcription-PCR using the primers listed in Supplementary Table 2. Mutants of these cDNAs were prepared by the QuikChange method, and the entire nucleotide sequences were verified. The cDNAs were ligated to the appropriate epitope sequences and cloned into the pcDNA3.1 (Invitrogen) and pGEX-6P-1 (GE Healthcare) vectors. For lentiviral transduction, pCSII-CMV-RfA-IRES-Blast (RIKEN BioResource Research Center) was used.

Antibodies

The following antibodies were used for immunoblotting. Catalogue numbers and dilutions are indicated in parentheses. P-IκBα (#9246; 1:1,000), IκBα (#4812; 1:1,000), P-p105 (#4806; 1:1,000), p105 (#13586; 1:1,000), P-p65 (#3033; 1:1,000), p65 (#8242; 1:1,000), p100/p52 (#4882; 1:1,000), P-RelB (#4999; 1:1,000), RelB (#4922; 1:1,000), P-JNK (#4668; 1:1,000), JNK (#5668; 1:1,000), P-IRF3 (#4947; 1:1,000), IRF3 (#4302; 1:1,000), P-TBK1 (#5483; 1:1,000), TBK1 (#3504; 1:1,000), P-IKKε (#8766; 1:1,000), IKKε (#2690; 1:1,000), IκBζ (#9244; 1:1,000), caspase 3 (#9662; 1:1,000), cleaved caspase 3 (#9661; 1:1,000), caspase 8 (#9746; 1:1,000), caspase 8 (#4790; 1:1,000) cleaved caspase 8 (#9496; 1:1,000), PARP (#9542; 1:1,000), P-RIP1 (#31122; 1:1,000), P-RIP3 (#57220; 1:1,000), RIP3 (#15828; 1:1,000), P-MLKL (#37333; 1:1,000), MLKL (#37705; 1:1,000), IRAK1 (#4504; 1:1,000), CYLD (#8462; 1:1,000), OTULIN (#14127; 1:1,000), KEAP1 (#8047; 1:1,000), AIFM1 (#5318, 1:1,000), Tom20 (#42406, 1:1,000), Lamin A/C (#4777, 1:1,000), and GST (#2622, 1:1,000) were obtained from Cell Signaling. HOIL-1L (sc-393754; 1:250), TNFR1 (sc-7895; 1:1,000), VCAM (sc-8304; 1:1,000), and β-actin (sc-47778; 1:250) were purchased from Santa Cruz Biotechnology. HOIP (ab125189; 1:1,000), NEMO (ab178872; 1:3,000), and K63-linkage specific ubiquitin (ab179434; 1:1,000) were purchased from Abcam. RIP1 (610458; 1:1,000) and FADD (610399; 1:1,000) were from BD Transduction Laboratories. OTUD1 (NBP1-

90484, Novus Biologicals; 1:1,000), tubulin (CLT9002, Cedarlane; 1:5,000), linear ubiquitin (Millipore, clone LUB9, MABS451; 1:1,000), SHARPIN (14626-1-AP, Proteintech; 1:5,000), KEAP1 (60027-1-Ig, Proteintech; 1:2,000), PGAM5(28445-1-AP, Proteintech; 1:1,000), DYKDDDDK (1E6, HRP-conjugate, Wako; 1:10,000), Myc (M192-7, HRP-conjugate, MBL; 1:20,000), and HA (11867423001, Roche; 1:2,000) were also used. To stimulate the lymphotoxin β receptor (LTβR), a 10:1 mix of the 4H8 WH2 (Adipogen, AG-20B-0008) and 3C8 (eBiosciences, 16-5671-82) monoclonal antibodies was used. For immunoprecipitation, Myc (sc-40, Santa Cruz Biotechnology; 1 μg), HA (16B12, BioLegend; 1 μg) and OTUD1 (NBP1-90484, Novus Biologicals; 3 μg) were used.

Cell culture and transfection

HEK293T, HeLa (ATCC), and MEF cells were cultured in DMEM, containing 10% fetal bovine serum, 100 IU/ml penicillin G, and 100 μg/ml streptomycin, at 37°C under a 5% CO_2 atmosphere. Bone marrow-derived macrophages (BMDM) were prepared basically as described[1]. Briefly, bone marrow cells were isolated from the femurs and tibias of B6 mice. After reticulocyte removal, the residual cells were cultured in 10% FCS/RPMI, supplemented with 5% of L-929 conditioned medium. The floating cells were removed,

and the attached cells were passed every 2 days. The resultant adherent cells were used as macrophages on day 7. To obtain splenic B cells, suspensions of mouse splenocytes were prepared from freshly removed mouse spleens, and erythrocytes were depleted with ACK lysing buffer (0.15 M NH_4Cl, 10 mM $KHCO_3$, 0.1 mM Na_2EDTA, pH 7.2), separated by BD IMag anti-mouse CD45R/B220 particles (BD Biosciences), and then maintained in RPMI containing 10% FBS, 55 μM 2-mercaptoethanol, and antibiotics, at 37 °C under a 5% CO_2 atmosphere. Plasmid transfection into HEK293T cells was performed using Lipofectamine 2000 (Thermo Fisher Scientific) or polyethylenimine (PEI). To stimulate the cells, poly(dA:dT) or poly(I:C) was transfected using Lipofectamine 2000 (Thermo Fisher Scientific). For the stable expression of FLAG-tagged OTUD1 in *Otud1*$^{-/-}$-MEFs, lentiviral infection followed by selection with 5 µg/ml blasticidin was performed.

Luciferase assay

HEK293T cells were cultured in 6-well or 24-well plates, and co-transfected with the pGL4.32 [*luc2P*/NF-κB-RE/Hygro] vector (Promega) and the phRL-TK *Renilla* Luciferase control reporter vector (Promega). At 24 h after transfection, the cells were lysed and the luciferase activity was measured with a GloMax 20/20 luminometer

(Promega), using the Dual-Luciferase Reporter Assay System (Promega).

Screening for DUBs

HEK293T cells cultured in 6-well plates were transfected with pcDNA3.1 plasmids encoding DUB (1 μg), HA-HOIP (30 ng), HOIL-1L-HA (15 ng), and HA-SHARPIN (10 ng), with NF-κB and control luciferase reporters. After 24 h, the luciferase activity was measured as described above. Taking the NF-κB-luciferase activity induced by LUBAC alone as 100%, the relative residual activity in the presence of the respective DUBs was calculated.

Gel filtration analysis

Cells were suspended in 50 mM Tris-HCl, pH 7.5, containing 1 mM $MgCl_2$, 1 mM DTT, 1 mM PMSF, and a protease inhibitor cocktail (Complete EDTA-free, Roche), and homogenized by a Dounce homogenizer. After adding an equal volume of buffer containing 300 mM NaCl, lysates were centrifuged at 100,000 *g* for 30 min. The supernatant was fractionated on an ÄKTA pure 25 chromatography system using a Superdex 200 HR (10/30) column (Cytiva), which was equilibrated with 50 mM Tris-HCl, pH 7.5, containing 150 mM NaCl. The column was calibrated by the elution profile

of a Gel Filtration HMW Calibration Kit (Cytiva).

Preparation of recombinant OTUD1

The pGEX-6P-1 expression vector, which encodes glutathione *S*-transferase (GST)-fused OTUD1-Wt, was expressed in *E. coli* BL21(DE3) (Novagen), and the recombinant protein was prepared as described previously[2].

In vitro DUB assay

For the *in vitro* DUB assay with diubiquitins, HEK293T cells were transfected with FLAG-OTUD1-Wt or catalytically inactive FLAG-OTUD1-CA and lysed in lysis buffer, containing 20 mM Tris-HCl, pH 7.5, 150 mM NaCl, 1% Triton X-100, and 1 mM DTT. The cell lysates were immunoprecipitated by a rabbit anti-FLAG antibody (Sigma-Aldrich, F7425) and protein A beads (Cytiva). After a wash with lysis buffer, the beads were reacted with 0.5 μg of M1-, K6-, K11-, K27-, K29-, K33-, K48- or K63-linked diubiquitins (Boston Biochem) in 20 mM Tris-HCl, pH 7.5, containing 1 mM DTT, for 30 min at 37 °C. For the *in vitro* deubiquitylation assay with polyubiquitin chains, the M1-, K48-, or K63-linked polyubiquitin chains (2.0 μg), prepared as described previously[3], were treated with 0.6 μM recombinant GST-OTUD1-Wt in a total volume of

20 µl of 20 mM Tris-HCl, pH 7.4, containing 1 mM DTT, for the indicated time periods at 37 °C.

Immunoprecipitation, SDS-PAGE, and immunoblotting

Cells were lysed with 50 mM Tris-HCl, pH 7.5, containing 150 mM NaCl, 1% Triton X-100, 2 mM PMSF, and complete protease inhibitor cocktail (Sigma). To detect covalent ubiquitination, cells were lysed with 1% SDS and heated at 95°C for 5 min. The denatured samples were diluted 10-fold with 50 mM Tris-HCl, pH 7.5, containing 150 mM NaCl and 1% Triton X-100. Immunoprecipitation was performed using appropriate antibodies followed by Protein G agarose beads (Cytiva) at 4°C with gentle rotation. Immunoprecipitates were washed five times with the lysis solution. Samples were separated by SDS-PAGE and transferred to PVDF membranes. After blocking, the membranes were incubated with the appropriate primary antibodies, followed by an incubation with HRP-conjugated secondary antibodies. The chemiluminescent images were obtained with an LAS4000 imaging analyzer (GE Healthcare) or a Fusion Solo S imaging system (Vilber). Uncropped immunoblotting images are shown in Supplementary Fig. 8.

Construction of *OTUD1*-knockout cells

To generate *OTUD1*-knockout HeLa and HEK293T cells, the gRNA cloning vector (Addgene; #41824) targeting 5′-CCGagaccggtgagtgccagccc-3′ and 5′-tacccggagttgctggccaTGG-3′ (Capital letters indicate PAM-sequence) in the coding sequence of human *OTUD1*, and the pCAG-hCas9 expression vector (Addgene; #51142) were transfected using Lipofectamine 3000 (Thermo Fisher). After 96 h, cells were cloned by limiting dilution to obtain single cell clones. The clones were validated by *Age*I or *Nco*I digestion, sequenced with PCR-amplified fragments to confirm mutations, and subjected to immunoblotting with an anti-OTUD1 antibody.

Construction of *Otud1*-knockout mice

C57BL/6J mice were used as the source of embryos for micromanipulation and subsequent breeding trials. A mixture of sgRNAs targeting the 5′-UTR of *Otud1*: 5′-attcgcggctcctgacgcggCGG-3′ (Capital letters indicate PAM-sequence) and 3′-UTR of *Otud1*: 5′-gaaccacacgaactctaattAGG-3′ (Capital letters indicate PAM-sequence), and Cas9 protein was injected into the cytoplasm of one cell-stage fertilized embryos. Zygotes that survived were transferred into the oviducts of pseudopregnant foster mothers. The heterozygous mutant mice were intercrossed to produce the homozygous mutant mice.

For PCR primers to detect the wild-type *Otud1* allele, we used 5′-GTATTTGGCTCAGTTGGCTC-3′ and 5′-TTCATACCGTGAGACATCAG-3′ oligo DNAs to detect the 450 bp band. For the *Otud1* deleted allele, we used 5′-ATCCGCGCGCTGCACTCCTG-3′ and 5′-TTCATACCGTGAGACATCAG-3′ oligo DNAs to detect the 440 bp band (which was 1,960 bp in the wild-type allele). C57BL/6J mice were kept in a specific pathogen-free, temperature-controlled facility at the Graduate School of Medicine, Osaka City University. Mice were euthanized according to the guidelines of the Animal Experiment Committee of Osaka City University, and all efforts were made to minimize the suffering of the animals. MEFs were prepared from littermate embryos (E12.5) of Wt- and *Otud1*-KO mice, transfected with an SV40 T-antigen containing pEF321-T plasmid, and then immortalized.

K63- and M1-TUBE pulldowns

Cells were lysed in RIPA buffer (50 mM Tris-HCl, pH 7.5, containing 150 mM NaCl, 1% NP-40, 1% sodium deoxycholate, and 0.1% SDS) supplemented with 2 mM PMSF, protease inhibitor cocktail, and 10 mM *N*-ethylmaleimide, and centrifuged at 20,000 *g* for 20 min at 4 °C. The cleared lysates (4 mg) were diluted 10-fold with 1% Triton X-100 buffer (50 mM Tris-HCl, pH 7.5, 150 mM NaCl, 1% Triton X-100), and then incubated

with 30 µl of K63-Tandem Ubiquitin Binding Entity (TUBE)-Agarose (LifeSensors, UM401), or 2.4 µg of K63-TUBE-Biotin (LifeSensors, UM304) or M1-TUBE-Biotin (LifeSensors, UM306) with 30 µl of Dynabeads M-280 Streptavidin (Veritas), overnight at 4°C. The precipitates were washed five times with 1% Triton X-100 buffer, boiled in SDS sample buffer, and then subjected to immunoblotting analysis.

qPCR

Cell lysis, reverse-transcription, and qPCR were performed with a SuperPrep Cell Lysis RT Kit for qPCR (TOYOBO) and Power SYBR Green PCR Master Mix (Life Technologies), according to the manufacturers' instructions. Quantitative real-time PCR was performed with a Step-One-Plus PCR system (Applied Biosystems) by the ΔΔCT method, using the following oligonucleotides: human *IL-6* sense, 5'-AGCCACTCACCTCTTC-3', and human *IL-6* anti-sense, 5'-GCCTCTTTGCTGCTTT-3'; human *ICAM1* sense, 5'-GTGGTAGCAGCCGCAGT-3' and human *ICAM1* anti-sense, 5'-TTCGGTTTCATGGGGGT-3'; human *BIRC3* sense, 5'-AGATGAAAATGCAGAGTCATCAAT-3' and human *BIRC3* anti-sense, 5'-CATGATTGCATCTTCTGAATGG-3'; and human *GAPDH* sense, 5'-

AGCAACAGGGTGGTGGAC-3' and human *GAPDH* anti-sense, 5'-GTGTGGTGGGGGACTGAG-3'.

For analyses with mouse-derived immune cells or tissues, total mRNA was extracted with an RNeasy Mini Kit (QIAGEN), and then transcribed to cDNA with ReverTra Ace qPCR RT Master Mix with gDNA Remover (TOYOBO), according to the manufacturers' instructions. Quantitative real-time PCR was performed with Power SYBR Green PCR Master Mix (Life Technologies) and a Step-One-Plus PCR system by the ΔΔCT method, using the following oligonucleotides: mouse *Ifnb1* sense: 5'-CCCTATGGAGATGACGGAGA-3' and mouse *Ifnb1* anti-sense: 5'-CTGTCTGCTGGTGGAGTTCA-3'; mouse *Isg15* sense: 5'-GGAACGAAAGCGGCCACAGCA-3' and mouse *Isg15* anti-sense: 5'-CCTCCATGGGCCTTCCCTCGA-3'; mouse *Isg56* sense: 5'-CCCTATGGAGATGACGGAGA-3' and mouse *Isg56* anti-sense: 5'-CTGTCTGCTGGTGGAGTTCA-3'; mouse *Cxcl10* sense, 5'-CCAAGTGCTGCCGTCATTTTC-3' and mouse *Cxcl10* anti-sense, 5'-GGCTCGCAGGGATGATTCAA-3'; mouse *Tnfaip3* sense, 5'-GCTTTCGCAGAGGCAGTAACAG-3' and mouse *Tnfaip3* anti-sense, 5'-AGCAAGTGCAGGAAAGCTGGCT-3'; mouse *Bcl-xl* sense, 5'-

GGTGAGTCGGATTGCAAGTT-3' and mouse *Bcl-xl* anti-sense, 5'-GCTGCATTGTTCCCGTAGAG-3'; mouse *Hmox-1* sense, 5'-AAGCCGAGAATGCTGAGTTCA-3' and mouse *Hmox-1* anti-sense, 5'-GCCGTGTAGATATGGTACAAGGA-3'; mouse *Nfe2l2* sense, 5'-TCTTGGAGTAAGTCGAGAAGTGT-3' and mouse *Nfe2l2* anti-sense, 5'-GTTGAAACTGAGCGAAAAAGGC-3'; mouse *Tnf* sense, 5'-TAGCCAGGAGGGAGAACAGA-3' and mouse *Tnf* anti-sense, 5'-TTTTCTGGAGGGAGATGTGG-3'; mouse *Il-1b* sense, 5'-CCCTGCAGCTGGAGAGTGTGGA-3' and mouse *Il-1b* anti-sense, 5'-TGTGCTCTGCTTGTGAGGTGCTG-3'; and mouse *Gapdh* sense, 5'-TTTGGCATTGTGGAAGGGCTCAT-3' and mouse *Gapdh* anti-sense, 5'-CACCAGTGGATGCAGGGATGATGT-3'.

Analyses of TNFR complexes I and II

The TNFR complex I analysis was performed as described previously[3]. Briefly, parental and *OTUD1*$^{-/-}$-HeLa cells were stimulated for the indicated times with 1 μg/ml FLAG-tagged TNF-α, and then lysed in 1 ml lysis buffer, containing 50 mM Tris-HCl, pH 7.5, 150 mM NaCl, 1% Triton X-100, and complete protease inhibitor cocktail. After

centrifugation at 20,000 *g* for 15 min, the supernatants were immunoprecipitated with 40 μl anti-FLAG M2 beads (Sigma) overnight at 4°C. For the TNFR complex II analysis, parental and *OTUD1*$^{-/-}$-HEK293T cells were treated with 10 ng/ml TNF-α and 10 μg/ml CHX. The cell lysates were then immunoprecipitated with an anti-FADD antibody (Santa Cruz; sc-271748) and immunoblotted.

RNA-seq

Parental and *OTUD1*$^{-/-}$-HeLa cells stimulated with 1 ng/ml IL-1β for 1 and 3 h, and *Otud1*$^{+/+}$- and *Otud1*$^{-/-}$-MEFs stimulated with 10 μg/ml poly(I:C) for 2 and 4 h, were lysed, and the total RNA was extracted using a RNeasy Mini kit (Qiagen, 74104) according to the manufacturer's instructions. The mRNA template library was constructed with a TruSeq Stranded mRNA LT Sample Prep Kit (Illumina), and the sequencing analysis was performed by Macrogen Corp. (Korea), using a NovaSeq 6000 sequencer (Illumina) and an S4 Reagent Kit. The data were processed by a Multidimensional Scaling Analysis and a Hierarchical Clustering Analysis by the Euclidean algorithm with the Complete Linkage program.

Cell survival assay

The number of viable cells was measured with a CellTiter-Glo Luminescent Cell Viability Assay (Promega), which quantifies the ATP content, and a trypan blue exclusion assay. The cell viability was also continuously monitored as an impedance-based cell index, using an xCELLigence RTCA S16 instrument (ACEA Biosciences Inc.). For the experiments, the parental and *OTUD1*$^{-/-}$-HEK293T cells (40,000 cells/well) or MEF cells (20,000 cells/well) were seeded in an E-Plate VIEW 16 (ACEA Biosciences, Inc.). The next day, the cells were stimulated with TC or TCZ, and the cell index was continuously monitored every 15 min. The data were analyzed with real-time cell analysis (RTCA) software, and normalized against the cell indices at the time of TC or TCZ treatment.

ROS measurement

Intracellular ROS levels were measured using an ROS Assay Kit -Highly Sensitive DCFH-DA (Dojindo), which is converted to the highly fluorescent 2′,7′-dichlorofluorescein (DCF) in the presence of an oxidant, according to the manufacturer’s instructions. Briefly, cells were cultured in 96-well black plates (clear bottom) and treated as indicated. After treatment, the cells were washed with HBSS (Gibco), and incubated with the DCFH-DA solution for 30 min at 37°C under a 5% CO_2 atmosphere. ROS production was analyzed with a Varioskan LUX multimode microplate reader (Thermo

Fisher Scientific) with excitation and emission wavelengths at 490 and 520 nm, respectively.

Identification of OTUD1-associated proteins by LC-MS/MS

An empty vector or a FLAG-tagged OTUD1 expressing vector was transfected into HEK293T cells in 10-cm dishes. At 24 h after transfection, the cells were washed with HEPES-saline (20 mM HEPES-NaOH, pH 7.5, 137 mM NaCl) and lysed with 20 mM HEPES-NaOH, pH 7.5, 150 mM NaCl, 1% Triton X-100, and complete protease inhibitor cocktail (Sigma). After centrifugation at 20,000 *g*, 4°C for 10 min, the supernatants were incubated with anti-FLAG M2 Magnetic Beads (M8823, Sigma) for 1 h at 4°C with gentle rotation. The beads were collected using a magnetic stand and washed four times with the 1% Triton X-100 lysis buffer, and then another three times with 50 mM ammonium bicarbonate. Proteins on the beads were digested with 200 ng trypsin/Lys-C mix (Promega, V5072) for 16 h at 37°C. The digests were reduced, alkylated, acidified with trifluoroacetic acid (Wako, 206–10731; TFA), and desalted using GL-Tip SDB (GL Sciences, 7820–11200). The eluates were evaporated in a SpeedVac concentrator and then dissolved in 0.1% TFA and 3% acetonitrile (ACN). The LC-MS/MS analysis of the resultant peptides was performed on an EASY-nLC 1200 UHPLC connected to an

Orbitrap Fusion mass spectrometer through a nanoelectrospray ion source (Thermo Fisher Scientific). The peptides were separated on a 75 µm inner diameter × 150 mm C18 reversed-phase column (Nikkyo Technos) with a linear 4–32% ACN gradient for 0–100 min, followed by an increase to 80% ACN for 10 min and a final hold at 80% ACN for 10 min. The mass spectrometer was operated in the data-dependent acquisition mode with a maximum duty cycle of 3 s. MS1 spectra were measured with a resolution of 120,000, an automatic gain control (AGC) target of 4e5, and a mass range from 375 to 1,500 *m/z*. HCD MS/MS spectra were acquired in the linear ion trap with an AGC target of 1e4, an isolation window of 1.6 *m/z*, a maximum injection time of 35 ms and a normalized collision energy of 30. Dynamic exclusion was set to 20 s. Raw data were directly analyzed against the SwissProt database restricted to *Homo sapiens*, using Proteome Discoverer version 2.4 (Thermo Fisher Scientific) with the Mascot search engine. The search parameters were as follows: (a) trypsin as an enzyme with up to one missed cleavage; (b) precursor mass tolerance of 10 ppm; (c) fragment mass tolerance of 0.6 Da; (d) carbamidomethylation of cysteine as a fixed modification; and (e) acetylation of protein N-terminus and oxidation of methionine as variable modifications. Peptides and proteins were filtered at a false discovery rate (FDR) of 1%, using the Percolator node and the Protein FDR Validator node, respectively. Label-free quantification was

performed based on the intensities of precursor ions, using the Precursor Ions Quantifier node. Normalization was performed such that the total sum of abundance values for each sample over all peptides was the same.

PRM analysis

To quantify KEAP1, AIFM1, CUL3, and PGAM5, two or three peptides/protein were measured by parallel reaction monitoring (PRM), an MS/MS-based targeted quantification method using high-resolution MS. The LC-MS/MS analysis was performed on an EASY-nLC 1200 UHPLC connected to a Q Exactive Plus mass spectrometer through a nanoelectrospray ion source (Thermo Fisher Scientific). Targeted HCD MS/MS scans were acquired by a time-scheduled inclusion list at a resolution of 70,000, an AGC target of 2e5, an isolation window of 2.0 *m/z*, a maximum injection time of 300 ms, and a normalized collision energy of 27. Time alignment and relative quantification of the transitions were performed using the Skyline software.

Sendai virus infection

MEFs and BMDMs were infected with Sendai virus (SeV) strain Cantell at a cell infectious unit (CIU) of 10 for 1 h. The washed cells were then incubated for the indicated times.

Dextran sulfate sodium (DSS)-induced IBD model

To analyze the DSS-induced colitis model, 8-week old Wt- and *Otud1*$^{-/-}$-C57/BL/6 background male mice were used. Each mouse was weighed once a day and monitored for the appearance of bloody and loose stools. Mice were sacrificed after a 7 day administration of 2.5% DSS (MW 36,000-50,000, MP Biomedicals) in drinking water. The stool consistency (diarrhea) and visible fecal blood were scored separately on a scale of 0 to 3, as described[4].

Acute hepatitis model mice

LPS with D-galactosamine (GalN)-induced acute hepatitis model mice were prepared by intraperitoneal injections of LPS (10 μg/kg) and GalN (800 mg/kg, Nacalai Tesque) in 200 μl PBS into 8-week old Wt- and *Otud1*$^{-/-}$-C57/BL/6 background mice. For the survival test, mice were injected with LPS/GalN and monitored every 15 min for 10 h. For immunoblotting or histopathologic examination, mice were sacrificed at 5 h after

injection, and liver tissues were lysed with RIPA buffer or fixed in a 4% paraformaldehyde (PFA) solution.

Sepsis model mice

LPS (20 mg/kg) was intraperitoneally injected into 8-week old Wt- and *Otud1*$^{-/-}$-C57/BL/6 background mice, and their survival was monitored.

Histopathological analysis

In the DSS-induced colitis model, colons and spleens were excised. Colons were distended in 0.9% saline and opened longitudinally, and then cut into three segments (proximal, middle, and distal colon). A transverse section of the spleen was made at the largest extension of the organ. After fixation in a 4% PFA solution, colon and spleen specimens were embedded in paraffin and processed for hematoxylin/eosin (H&E) and immunohistochemical staining. The histologic scores of colitis were evaluated using a quantitative evaluation form, as described[5].

In the sepsis model or acute hepatitis model, a total of 5 sections of liver tissue (one section each from the left lateral lobe, left middle lobe, right middle lobe, right lateral

lobe and caudate lobe) were fixed in a 4% PFA solution, embedded in paraffin, and processed for H&E and immunohistochemical staining.

Immunohistochemical analysis

Paraffin sections (4-µm thickness) were deparaffinized and dehydrated through a graded ethanol series. 8-Hydroxy-2-deoxy-guanosine (8-OHdG) staining was performed using a Histofine MOUSESTAIN kit (Nichirei, 414321) and an anti-8-OHdG (8-hydroxy-2'-deoxyguanosine) antibody (JaICA, MOG-100P) diluted 1:50. To detect cell death (necrosis and/or apoptosis), TUNEL staining was performed using an ApopTag® Peroxidase *In Situ* Apoptosis Detection Kit (Millipore, S7100), according to the manufacturer's instructions. TUNEL-positive cells in the spleens were counted in six 40x fields of each specimen, and quantified as the number of positive cells per field. Hepatocellular death was scored on a semiquantitative scale of 1 to 3, using the following criteria: 1, ≤1% TUNEL-positive cells; 2, >1% but ≤5% TUNEL-positive cells; 3, >5% TUNEL-positive cells. Microscopic image capture was performed using an Olympus BX53 microscope equipped with a DP72 digital microscope camera and a DP2-BSW image acquisition system (Olympus Corp., Tokyo, Japan).

NF-κB p65 staining was performed using the NF-κB p65 (D14E12) XP® Rabbit monoclonal antibody (#8242, Cell Signaling), diluted 1:800. Immunoreactivity was detected using a VECTASTAIN Elite ABC kit for rabbit (Vector Laboratories, Rabbit IgG, #PK-6101) and 3,3'-diaminobenzidine tetrahydrochloride (Dojindo Molecular Technologies). As all specimens showed either less than 1% or more than 50% positive nuclear staining of NF-κB p65 in hepatocytes, the extents were scored on a semiquantitative scale of 0 to 2 (0: not detectable, 1: less than 1%, 2: more than 50%).

Gene expression database analysis

The correlation of patient survival and the level of OTUD1 gene expression, and the OTUD1 gene comparison in normal or tumour kidney cortex, were analyzed online using GEPIA (http://gepia.cancer-pku.cn/). For the analysis of gene expression correlation, TCGA tumour (KIRC), TCGA normal (KIRC), and GTEx (Kidney-Cortex) were selected.

Statistics. One-way ANOVA followed by a post-hoc Tukey HSD test, *t*-test, Mann-Whitney test, and Log-rank test of Kaplan-Meier survival curve were performed using the GraphPad Prism 8 software. For all tests, a P value of less than 0.05 was considered statistically significant. For animal studies, no randomization was performed.

SUPPLEMENTARY REFERENCES

1. Weischenfeldt J, Porse B. Bone Marrow-Derived Macrophages (BMM): Isolation and Applications. *CSH Protoc* 2008, **2008:** pdb prot5080.

2. Oikawa D, Shiota M, Goto E, Komakura K, Wanibuchi H, Tokunaga F. Generation of Rat Monoclonal Antibodies Against a Deubiquitinase, Ovarian Tumor Domain-Containing Protein 1. *Monoclon Antib Immunodiagn Immunother* 2018, **37**(4)**:** 180-184.

3. Tokunaga F, Nishimasu H, Ishitani R, Goto E, Noguchi T, Mio K*, et al.* Specific recognition of linear polyubiquitin by A20 zinc finger 7 is involved in NF-kappaB regulation. *EMBO J* 2012, **31**(19)**:** 3856-3870.

4. Melgar S, Karlsson A, Michaelsson E. Acute colitis induced by dextran sulfate sodium progresses to chronicity in C57BL/6 but not in BALB/c mice: correlation between symptoms and inflammation. *Am J Physiol Gastrointest Liver Physiol* 2005, **288**(6)**:** G1328-1338.

5. Dieleman LA, Palmen MJ, Akol H, Bloemena E, Pena AS, Meuwissen SG*, et al.* Chronic experimental colitis induced by dextran sulphate sodium (DSS) is characterized by Th1 and Th2 cytokines. *Clin Exp Immunol* 1998, **114**(3)**:** 385-391.

Supplementary Table 1 Histopathological changes in the livers of mice 24 h after LPS treatment.

	LPS	Inflammatory cell infiltration	Fatty change	Hydropic degeneration	Midzonal hepatocellular necrosis
Male					
Otud1$^{+/+}$	-	0/3	0/3	0/3	0/3
	+	6/6	6/6	2/6	0/6
Otud1$^{-/-}$	-	0/3	0/3	0/3	0/3
	+	6/6	6/6	0/6	0/6
Female					
Otud1$^{+/+}$	-	0/3	0/3	0/3	0/3
	+	6/6	0/6	4/6	2/6
Otud1$^{-/-}$	-	0/3	0/3	0/3	0/3
	+	6/6	0/6	3/6	5/6

Supplementary Table 2 Primers used to amplify human DUBs.

No.	DUB	forward primer	reverse primer
1	USP1	CGCGGATCCATGCCTGGTGTCATACCTAG	CCGCTCGAGTTATAATTTCTTATAAAATA
2	USP2	CGCGATATCATGTCCCAGCTCTCCTCCAC	CCGCTCGAGTTACATTCGGGAGGGCGGGC
3	USP3	CGCGGATCCATGGAGTGTCCACACCTGAG	CCGCTCGAGTTAAAGTTTATCCGATCCAG
4	USP4	CGCGGATCCATGGCGGAAGGTGGAGGCTG	CCGCTCGAGTTAGTTGGTGTCCATGCTGC
5	USP5	CGCGGATCCATGGCGGAGCTGAGTGAGGA	CCGCTCGAGTTAGCTGGCCACTCTCTGGT
6	USP6	CGCGGATCCATGGACATGGTAGAGAATGC	CCGCTCGAGTTACTGTAACATAGAGTACT
7	USP7	CGGGATCCGCATGAACCACCAGCAGCAGCAGCA	CCGCTCGAGTCAGTTATGGATTTTAATGGCCT
8	USP8	CGCGGATCCGCCGCCATGCCTGCTGTGGCT	CCGCTCGAGTTATGTGGCTACATCAGTTA
9	USP9X	CGCGGATCCATGACAGCCACGACTCGTGG	CTAGTCTAGATTATTGATCCTTGGTTTGAG
10	USP9Y	TTTGCGGCCGCTCATGACAGCCATCACTCATGGCT	AAAGGGCCCTCACTGATCCTTCATCTGAGGTGA
11	USP10	AAGGAAAAAAGCGGCCGCGCCGCCATGGCCCTCCACAGC	CTAGTCTAGATTACAGCAGGTCCACTCGGC
12	USP11	CCGGAATTCATGGCAGTAGCCCCGCGACT	CCGCTCGAGTTAATTAACATCCATGAACT
13	USP12	CGCGGATCCATGGAAATCCTAATGACAGT	CCGCTCGAGTTAGTCCCGAGACTGATAGA
14	USP13	AAGGAAAAAAGCGGCCGCATGCAGCGCCGGGGCGC	CCGGGGCCCTTAGCTTGGTATCCTGCGGT
15	USP14	CGCGGATCCATGCCGCTCTACTCCGTTAC	CCGGAATTCTTACTGTTCACTTTCCTCTT
16	USP15	AAGGAAAAAAGCGGCCGCATGGCGGAAGGC	CCGGGGCCCTTAGTTAGTGTGCATACAGT
17	USP16	CGCGGATCCATGGGAAAGAAACGGACAAA	CCGCTCGAGTTACAGTATTCTCTCATAAA
18	USP17	CGCGGATCCATGGAGGACGACTCACTCTA	CCGGAATTCTTACTGGCACACAAGCAGAG
19	USP18	CGCGGATCCATGAGCAAGGCGTTTGGGCT	CCGCTCGAGTTAGCACTCCATCTTCATGT
20	USP19	CCGGAATTCATGTCTGGCGGGGCCAGTGC	CCGCTCGAGTTATCTCCAGCGACTCTGGG
21	USP20	CGCGGATCCATGGGGGACTCCAGGGACCT	CCGGAATTCTTACACGGCCCGCGTCTCGG
22	USP21	CCGGAATTCATGCCCCAGGCCTCTGAGCA	CCGCTCGAGTTACAGGCACCGGGGTGGCT
23	USP22	CGCGGATCCATGGTGTCCCGGCCAGAGCC	CCGGAATTCTTACTCGTATTCCAGGAACT
24	USP24	CCGCGATATCATGGAATCGGAGGAGGAGCA	AAGGAAAAAAGCGGCCGCCTAGGGATCAACATCATCAA
25	USP25	CGCGGATCCATGACCGTGGAGCAGAACGT	CCGCTCGAGTTATCTTCCATCAGCAGGAG
26	USP26	CGCGGATCCATGGCTGCCCTATTCCTACG	CCGCTCGAGTTATTCCTTCTGAAGGGTCT
27	USP27	CGCGGATCCATGTGTAAGGACTATGTATA	CCGGAATTCTTAGTAGGCTTGTGTGTTCA
28	USP28	CGCGGATCCATGACTGCGGAGCTGCAGCA	AAGGAAAAAAGCGGCCGCTTATTTCACTGT
29	USP29	AAGGAAAAAAGCGGCCGCATGATATCTCTAAAGGT	CCGGGGCCCTTAAGCAGGTCTGTACAAAG
30	USP30	CGCGGATCCATGCTGAGCTCCCGGGCCGA	CCGGAATTCTTATTCTTCAGACTTGCACT
31	USP31	CGCGGATCCATGTCCAAGGTAACGGCGCC	CCGCTCGAGTCACTGAGGTTTTTGAGAAG
32	USP32	AAGGAAAAAAGCGGCCGCATGGGTGCCAAGGAGTC	CCGGGGCCCTTACTGTAACACACAGTACT
33	USP33	CGCGGATCCATGACAGGATCAAATTCACA	AAGGAAAAAAGCGGCCGCTTACAAAGACCGAGTTT
34	USP34	AAGGAAAAAAGCGGCCGCATGTGCGAGAACTGCGC	CCGGGGCCCTTAAGAAGCAGCTTGGTTTC
35	USP35	CGCGGATCCGCCGCCATGGACAAGATCTTG	CCGGAATTCTTAGAAGACCAGTCTGTGGAA
36	USP36	CGCGGATCCATGCCAATAGTGGATAAGTT	CCGCTCGAGTTAGCGGCGATAGCTGAGGC
37	USP37	AAGGAAAAAAGCGGCCGCATGTCTCCTCTGAAGAT	CCGGGGCCCTTACGAGGCCTGACGGGTAG
38	USP38	CCGGAATTCATGGACAAGATCCTGGAGGG	AAGGAAAAAAGCGGCCGCTTAAAATACGAGTCTGC
39	USP39	CCGGAATTCATGTCCGGCCGGTCTAAGCG	CCGCTCGAGTTAAGCCCCCTGCTGGTTGG
40	USP40	CGCGGATCCATGTCACTTTTTTTAAGGGT	AAGGAAAAAAGCGGCCGCTTATCTGAAGCTCCCCA
41	USP42	CGCGATATCGCCGCCATGACCATAGTTGAC	CCGCTCGAGTCAATCACCCTGGCCATACT
42	USP43	CCGCGATATCATGGACCTGGGCCCCGGGGA	CTAGTCTAGATTAAAAGCTGGACTCCGGTA
43	USP44	CGCGGATCCATGCTAGCAATGGATACGTG	CCGCTCGAGTTAGCTAAGGATTTCATTAG
44	USP45	CCGCTCGAGGGCCGCCATGCGGGTGAAAGA	CGCGGGCCCTTATAATACTCTTTCATAGA
45	USP46	CGCGGATCCATGACTGTCCGAAACATCGC	CCGCTCGAGTTACTCTCTTGACTGATAGA
46	USP47	CGCGATATCATGGTGCCCGGCGAGGAGAA	CCGCTCGAGTTAGTCTTGAGTCAGATCTTTAT
47	USP48	CGCGGATCCATGGCCCCGCGGCTGCAGCT	CCGGAATTCTTAATGTCCAAGAAGACCAG
48	USP49	CCGCGATATCATGGATAGATGCAAACATGT	CTAGTCTAGATTAACCCCTTTCCGTGTCCC
49	USP51	CCGGAATTCATGGCCCAGGTTCGAGAAAC	CCGCTCGAGTTAGTCTTTCTCTAGACCCT
50	USP52	CCGGAATTCATGAACTTTGAGGGTCTGGA	CCGCTCGAGTTAGAGCGCCAGCACTGAGG
51	USP53	AAGGAAAAAAGCGGCCGCATGGCATGGGTAAAATT	CCGGGGCCCTTAAGATAGTGAATTATTAC
52	CYLD	CGCGGATCCGCATGAGTTCAGGCTTATGGAG	AAGGAAAAAAGCGGCCGCTTATTTGTACAAACTCATTG
53	USPL1	CGCGGATCCGCCGCCATGATGGATTCTCCG	CCGCTCGAGTCAATAATTCTCAAACAGA
54	UCHL1	CGCGGATCCATGCAGCTCAAGCCGATGGA	CCGCTCGAGTTAGGCTGCCTTGCAGAGAG
55	UCHL3	CCGGAATTCATGGATCCTGAACTCCTTAG	CCGCTCGAGTTATGCTGCAGAAAGAGCAA
56	UCHL5	CGCGGATCCATGACGGGCAATGCCGGGGA	CCGGAATTCTTATTTGGTTTCCTGAGCTT
57	BAP1	CGCGGATCCGCATGAATAAGGGCTGGCTGGA	CCGCTCGAGTCACTGGCGCTTGGCCTTGT
58	ATXN3	CGGGATCCGCATGGATTTCATCTTTCATGAGA	CCGCTCGAGTTATTTTTCCCCTTTGATTTTCA
59	AXTN3L	CGGGATCCGCATGGATTTCATCTTTCATGAGA	CCGCTCGAGTTATTTTTCCCCTTTGATTTTCA
60	JOSD1	CGCGGATCCATGAGTTGTGTGCCATGGAA	CCGGAATTCTTACACATCGGTCCTCCAAC
61	JOSD2	CGCGGATCCATGTCCCAGGCCCCGGGAGC	CCGGAATTCTTAGTCTGTCCGCAGCCAGC
62	OTUB1	CGCGGATCCGCCACCATGGCGGCGGAGGAA	CCGCTCGAGCTATTTGTAGAGGATATCGT
63	OTUB2	CGCGGATCCGCCACCATGAGTGAAACATCT	CCGGAATTCTCAATGTTTATCGGCTGCA
64	OTUD1	CCGGAATTCATGCAGCTCTACAGCAGCGTCTGCA	CCGCTCGAGTCAAGAGCATGCATTTTGTTCA
65	OTUD2	CGGGATCCGCATGTTTGGCCCCGCTAAAGGT	CCGCTCGAGTCACACTTCTCCAAAGTTGGT
66	OTUD3	CGCGGATCCGCATGTCCCGAAAGCAGGCGGC	CCGCTCGAGTCAGATGTTGAGAGCGGCGA
67	OTUD4	CGCGGATCCATGGCCTGTATTCACTATCT	CCGCTCGAGTCAAGTGTGCTGTCCCCTAT
68	OTUD5	CGCGGATCCGCATGACTATACTCCCCAAAAAG	CCGCTCGAGTCAACTCTTGTCTGGGGGCG
69	OTUD6A	CGGGATCCGCATGGATGATCCGAAGAGTGAACA	TAAAGCGGCCGCCTACAGGAGACGCGGGAGCA
70	OTUD6B	CGGGATCCGCATGGAGCCCCGGGTGAGGGTTGA	CCGCTCGAGTTAGCTGCAATTTTCAGTAACT
71	A20	CCGGAATTCATGGCTGAACAAGTCCTTCC	AAGGAAAAAAGCGGCCGCTTAGCCATACATCTGCTTGA
72	OTUD7B	CCGGAATTCATGACCCTGGACATGGATGC	CTAGTCTAGATCAGAACCTGTGCACCAGGA
73	OTUD7A	CCGGAATTCCGCCGCCATGGTTTCTAGTGT	CTAGTCTAGATTAGGGCCGGGCCCCGCGCG
74	TRABID	CCGGAATTCATGTCAGAACGTGGAATTAA	CTAGTCTAGATTATTCATCTTCATCATCCT
75	VCPIP1	AAGGAAAAAAGCGGCCGCGCCGCCATGTCTCAGCCGCCG	CTAGTCTAGATTAAGAGTGATCCATTGGCT
76	OTULIN	CGCGGATCCGCATGAGTCGGGGGACTATGCC	CTAGTCTAGATTATAGACTGGTCTCCTCACACA
77	ALG13	AAGGAAAAAAGCGGCCGCATGAAGTGCGTGTTTGTTAC	CTAGTCTAGATTACATACCCTGAGGTACAT
78	BRCC36	CCGGAATTCATGGCGGTGCAGGTGGTGCA	CCGGGGCCCTTATTCTAGAGAAGAAAGTT
79	CSN5	CGCGGATCCATGGCGGCGTCCGGGAGCGG	CCGGAATTCTTAAGAGATGTTAATTTGAT
80	PSMD14	CCGGAATTCATGGACAGACTTCTTAGACT	CTAGTCTAGATTATTTAAATACGACAGTAT
81	STAMBP	AAGGAAAAAAGCGGCCGCGCCGCCATGTCTGACCATGGA	CTAGTCTAGATTATCGAAGGTCTGTGATGGT
82	AMSH-LP	AAAGATATCATGGATCAGCCTTTTACTGTGAATTC	TAAAGCGGCCGCTCACCTCAGATCCAACACAATTA
83	MPND	CGCGGATCCATGGCAGCTCCGGAGCCGCT	CCGCTCGAGTCAGCTCCCCTGCTTGAGGA
84	MYSM1	AAGGAAAAAAGCGGCCGCATGGCGGCTGAAGAGGC	CCGGGGCCCTTACATTAACAATTCCTTTG
85	EIF3H	CGGGATCCGCATGGCGTCCCGCAAGGAAGG	CCGCTCGAGTTAGTTGTTGTATTCTTGAA
86	CSN6	CCGGAATTCATGGCGGCGGCGGCGGCGGC	CTAGTCTAGATCAGAAAAAGAGCCCGCGCA
87	PSMD7	CGCGGATCCATGCCGGAGCTGGCAGTGCA	CCGCTCGAGTTACTTTTTCTCCTTTTTCTCCTC
88	EIF3F	CGCGGATCCATGGCCACACCGGCGGTACC	CCGCTCGAGTCACAGGTTTACAAGTTTTTCATT

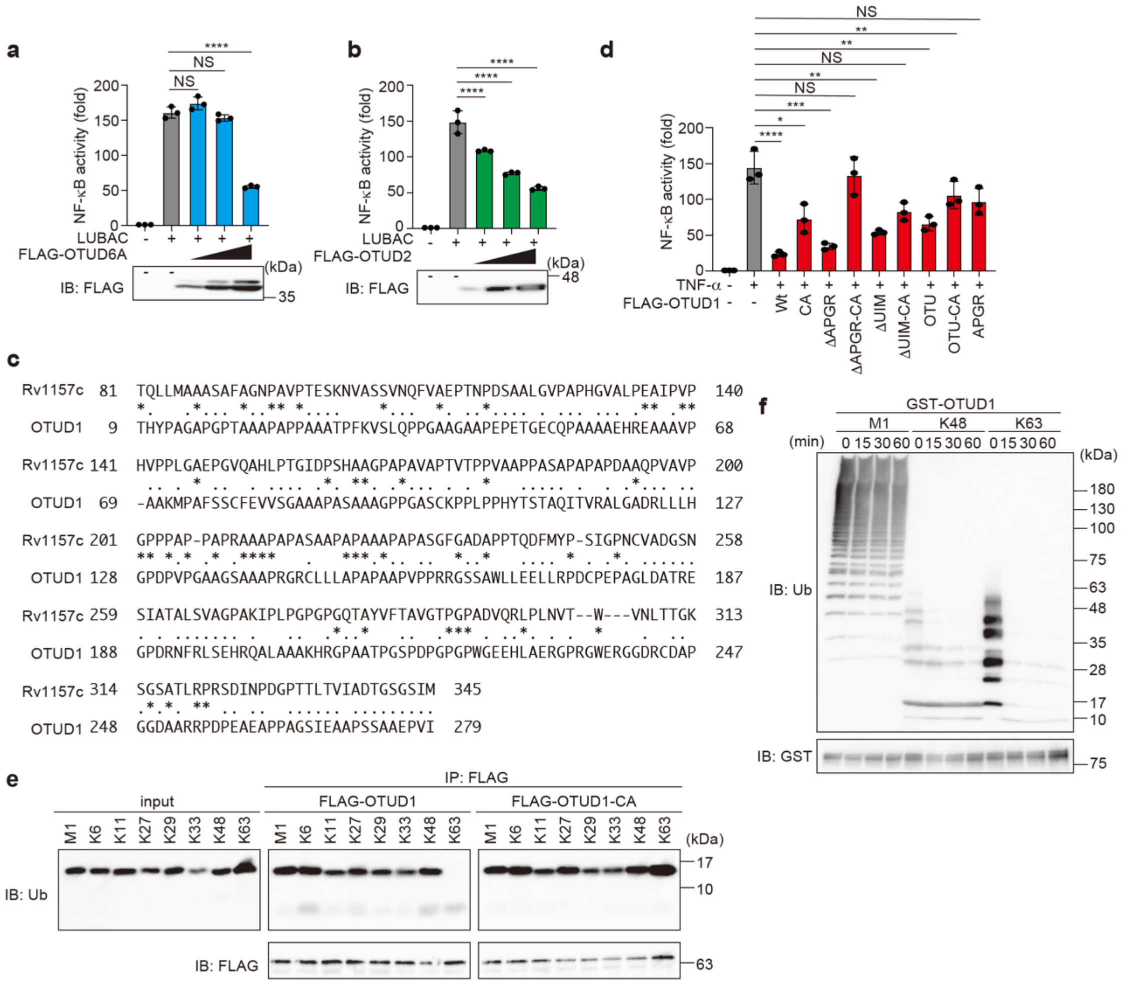

Supplementary Fig. 1 Characterization of LUBAC-suppressive DUBs, including OTUD1. a, b, OTUD6A and OTUD2 suppress LUBAC activity. The effects of increasing amounts (0.1, 0.3, and 1.0 μg) of OTUD6A (**a**) or OTUD2 (**b**) on LUBAC-induced NF-κB activity were analyzed, as in Fig. 1b. **c,** Amino acid sequence alignment of hypothetical protein Rv1157c in *Mycobacterium tuberculosis* and the N-terminal region of OTUD1. Identical and chemically similar residues are denoted by *asterisks* and *dots*, respectively. **d,** Effect of Wt- and mutants of OTUD1 on the TNF-α-induced NF-κB activity. A similar analysis to that in Fig. 1d was performed by stimulating cells with 10 ng/ml TNF-α for 6 h. **a, b, d,** Data are shown as mean ± SD by ANOVA post-hoc Tukey test (n = 3). *: P<0.05, **: P<0.01, ***: P<0.001, ****: P<0.0001, NS: not significant. **e,** OTUD1 hydrolyzes K63-diubiquitin. Wt or the active-site mutant of OTUD1 was expressed in HEK293T cells, and immunoprecipitated with anti-FLAG antibody-immobilized beads. Various diubiquitins were then treated with the immunoprecipitates for 1 h, and the cleavage of diubiquitin was investigated by

immunoblotting with the indicated antibodies. **f**, OTUD1 cleaves K63-linked polyubiquitin chains. *In vitro* DUB assays were performed using recombinant GST-OTUD1 and M1-, K48-, and K63-linked polyubiquitin chains. Samples were incubated for the indicated periods and immunoblotted with the indicated antibodies.

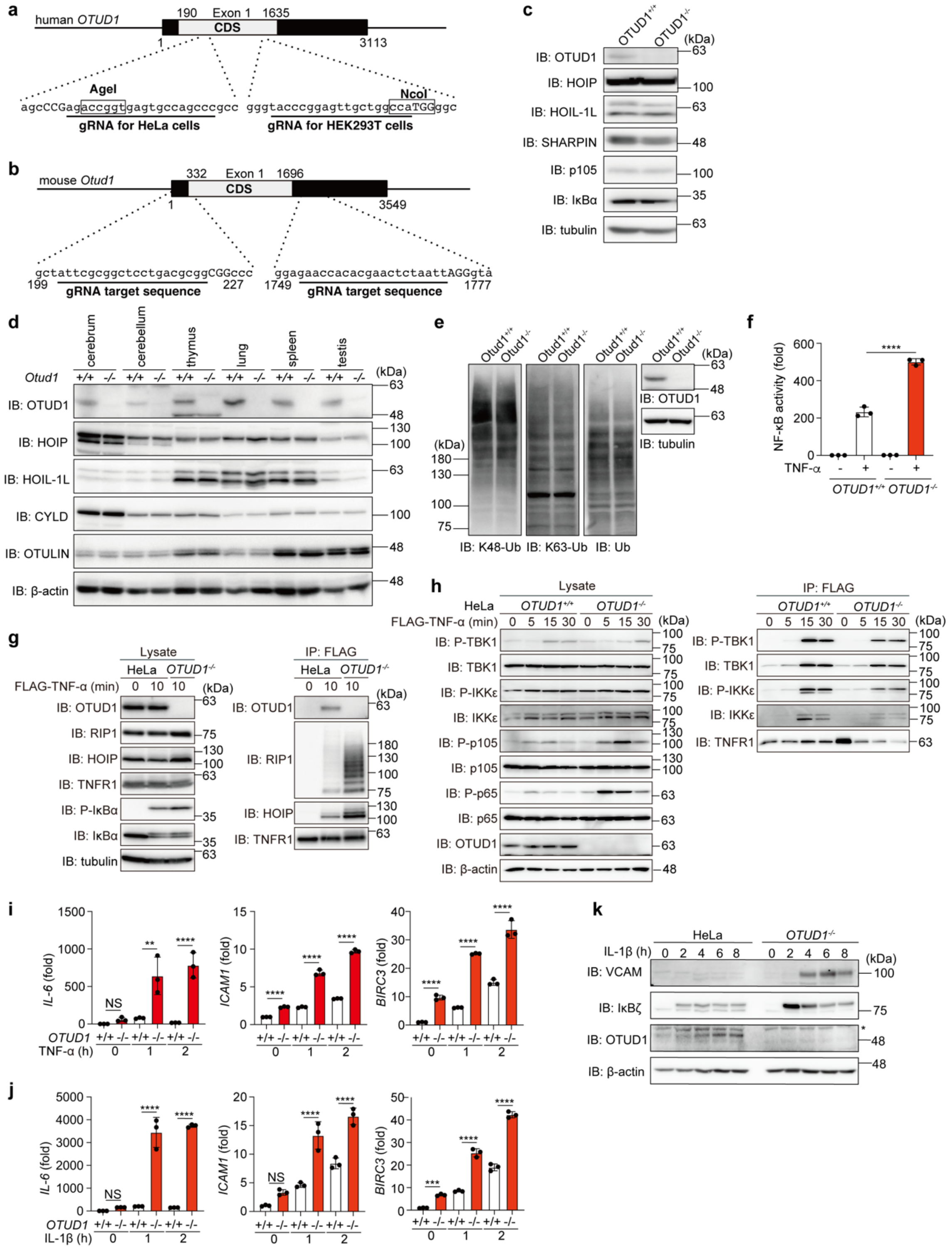

Supplementary Fig. 2 Construction of *OTUD1*-deficient cells and mice, and their effects on inflammatory cytokine-induced NF-κB activation. a, Scheme for *OTUD1*-KO targeting. Two independent gRNAs were used to target

exon 1 of the *OTUD1* gene in HeLa and HEK293T cells. **b**, Scheme for *Otud1*-KO targeting in mouse. Two gRNAs were used to delete the entire exon 1 of the *Otud1* gene. **c**, Immunoblotting of parental and *OTUD1*$^{-/-}$-HEK293T cells. The cell lysates were immunoblotted with the indicated antibodies. **d**, Expression of OTUD1 in various mouse tissues. Immunoblotting analyses of lysates from various *Otud1*$^{+/+}$- and *Otud1*$^{-/-}$-mouse tissues were performed. **e**, Ubiquitin dynamics were not changed by the genetic ablation of *Otud1*. Cell lysate from *Otud1*$^{+/+}$- and *Otud1*$^{-/-}$-MEFs were immunoblotted by the indicated antibodies. **f**, Enhanced TNF-α response in *OTUD1*-deficient cells. The NF-κB luciferase reporter was transfected into parental- and *OTUD1*$^{-/-}$-HEK293T cells, and luciferase activity was analyzed after 6 h stimulation with 60 ng/ml TNF-α. **g,** OTUD1 is a component of TNFR signaling complex I. A similar analysis to that in Fig. 2a was performed, and samples were immunoblotted with the indicated antibodies. **h,** Reduced TNF-α-mediated recruitment and activation of TBK1/IKKε to TNFR. A similar analysis as in Fig. 2a was performed, and samples were immunoblotted with the indicated antibodies. **i, j,** Enhanced expression of NF-κB target genes in inflammatory cytokine-treated *OTUD1*$^{-/-}$-HeLa cells. Parental and *OTUD1*$^{-/-}$-HeLa cells were stimulated with 10 ng/ml TNF-α (**i**) or 1 ng/ml IL-1β (**j**) for the indicated periods, and a qPCR analysis was performed. **f**, **i**, **j**, Data are shown as mean ± SD by ANOVA post-hoc Tukey test (n = 3). **: P<0.01, ***: P<0.001, ****: P<0.0001, NS: not significant. **k,** Increased expression of NF-κB-target proteins in *OTUD1*$^{-/-}$-HeLa cells. Cells were stimulated with 1 ng/ml IL-1β for the indicated periods, and cell lysates were analyzed by immunoblotting. *: nonspecific signal.

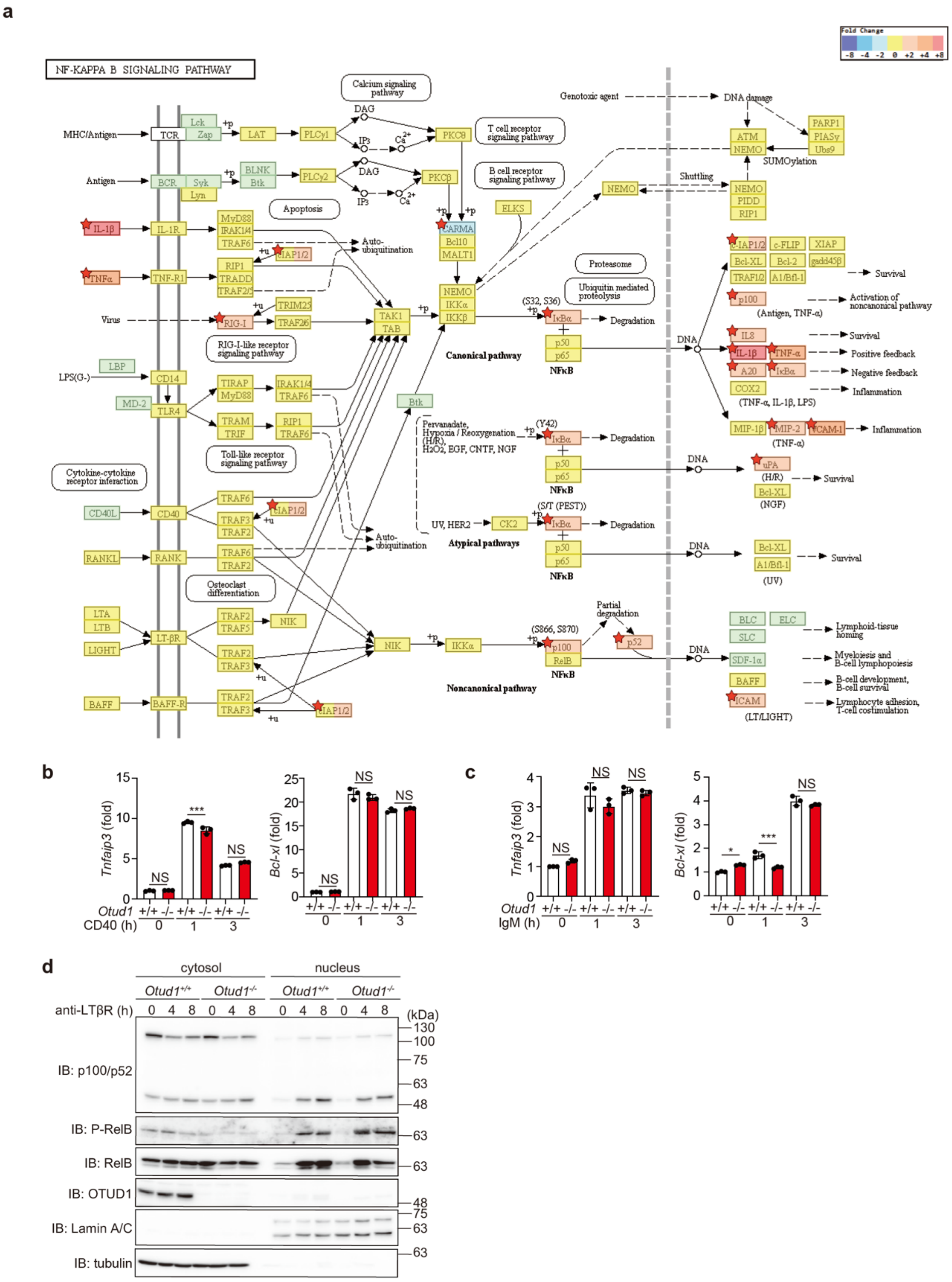

Supplementary Fig. 3 Effect of OTUD1 on NF-κB signaling pathways. a, Enhanced expression of NF-κB target genes in IL-1β-treated *OTUD1*$^{-/-}$ cells. The KEGG NF-κB signaling pathway map of various genes in IL-1β-treated parental and *OTUD1*$^{-/-}$-HeLa cells is shown. **b**, **c**, The genetic deficiency of

Otud1 does not affect CD40- and B cell receptor-mediated NF-κB activation. Splenic B cells from *Otud1*$^{+/+}$- and *Otud1*$^{-/-}$-mice were stimulated with 3 μg/ml CD40L (**b**) or 5 μg/ml IgM (**c**) for the indicated periods, and qPCR analyses were performed. Data are shown as mean ± SD by ANOVA post-hoc Tukey test (n = 3). *: P<0.05, ***: P<0.001, NS: not significant. **d**, OTUD1 is not involved in the non-canonical NF-κB pathway. *Otud1*$^{+/+}$- and *Otud1*$^{-/-}$-MEFs were stimulated with 0.3 μg/ml anti-lymphotoxin β receptor (LTβR) antibodies for the indicated periods. Cell lysates were separated into cytosolic and nuclear fractions, and immunoblotted with the indicated antibodies.

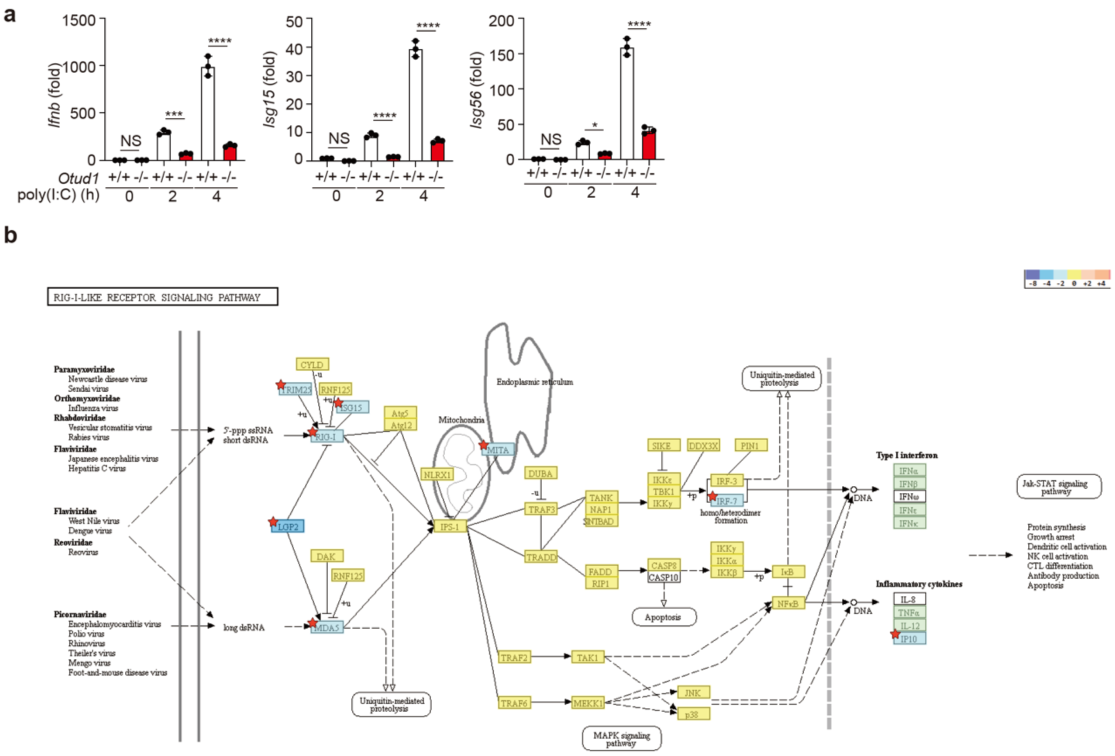

Supplementary Fig. 4 Reduced antiviral signaling in *Otud1*$^{-/-}$-MEFs. a, *Otud1*$^{+/+}$- and *Otud1*$^{-/-}$-MEFs were stimulated with 10 µg/ml poly(I:C) for the indicated periods, and qPCR was performed. Data are shown as mean ± SD by ANOVA post-hoc Tukey test (*n* = 3). *: *P*<0.05, ***: *P*<0.001, ****: *P*<0.0001, NS: not significant. **b,** Reduced IFN antiviral signaling in *Otud1*$^{-/-}$-MEFs. KEGG pathway maps on the RIG-I-like receptor signaling pathway are indicated.

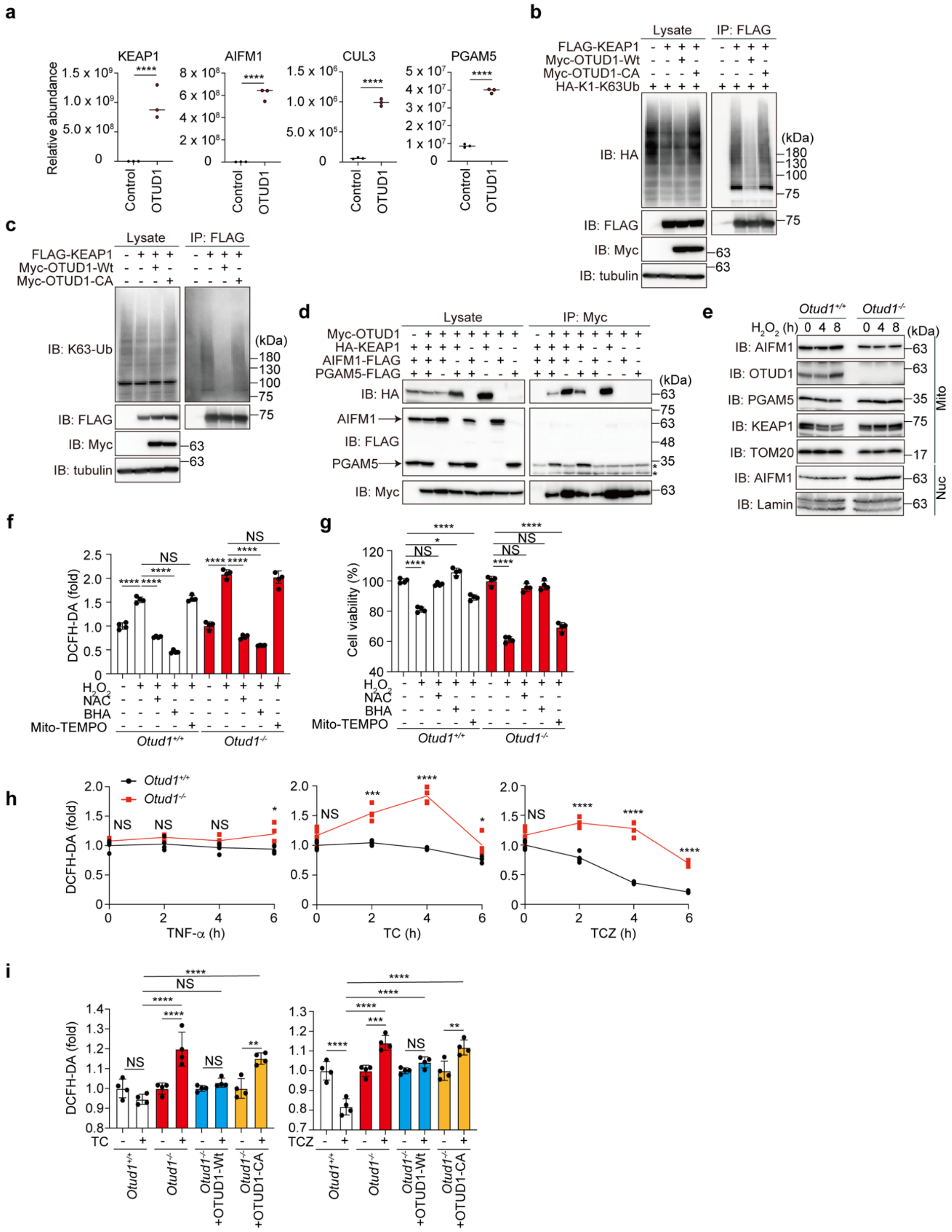

Supplementary Fig. 5 OTUD1 regulates ROS production and cell death pathways. a, The amounts of proteins co-precipitated with FLAG-OTUD1 were quantified by targeted mass spectrometry, using the parallel reaction monitoring (PRM) method. **b, c,** OTUD1 removes the K63-ubiquitin chain from KEAP1. FLAG-KEAP1, Myc-OTUD1-Wt/-CA, and HA-K1-K63-Ub, which has all of the

Lys residues in ubiquitin except for Lys63, were replaced with Arg, were transfected to *OTUD1*$^{-/-}$-HEK293T cells as indicated. The cells were then lysed with 1% SDS and heat denatured at 95°C, and the lysate and FLAG-immunoprecipitates were blotted with the depicted antibodies. **d**, OTUD1 binds PGAM5 *via* KEAP1. Myc-OTUD1, HA-KEAP1, AIFM1-FLAG, and PGAM5-FLAG were expressed in HEK293T cells as indicated. The cell lysates and anti-Myc immunoprecipitates were immunoblotted with the indicated antibodies. **e**, Intranuclear localization of AIFM1 in *Otud1*$^{-/-}$-MEFs. MEFs were treated with 0.3 mM H_2O_2 for the indicated periods, and fractionated into mitochondrial (Mito) and nuclear (Nuc) fractions. The samples were then immunoblotted with the indicated antibodies. **f**, **g**, Broad antioxidant scavengers prevent ROS production and recover cell viability. *Otud1*$^{+/+}$- and *Otud1*$^{-/-}$-MEFs were treated with 0.3 mM H_2O_2, 10 mM NAC, 10 μM BHA, and 3 μM Mito-TEMPO, as indicated for 2 h (**f**) or 8 (**g**) h. The ROS production (**f**) and cell viability (**g**) were then analyzed. **h**, Enhanced ROS generation in *Otud1*$^{-/-}$ cells upon apoptotic and necroptotic stimuli. *Otud1*$^{+/+}$- and *Otud1*$^{-/-}$-MEFs were treated with 10 ng/ml TNF-α, 10 μg/ml CHX, and/or 20 μM ZVAD as indicated, and the intracellular ROS levels were analyzed by a DCFH-DA assay. **i**, Catalytic activity of OTUD1 protects cells from ROS generation upon apoptotic and necroptotic stimuli. A similar analysis as in Fig. 5l was performed after TC or TCZ stimulation for 2 h. Data are shown as mean ± SD by *t*-test (**a**; n = 3, **h**; n = 4) or ANOVA post-hoc Tukey test (**f**, **g, i**; n = 4). *: P<0.05, **: P<0.01, ***: P<0.001, ****: P<0.0001, NS: not significant.

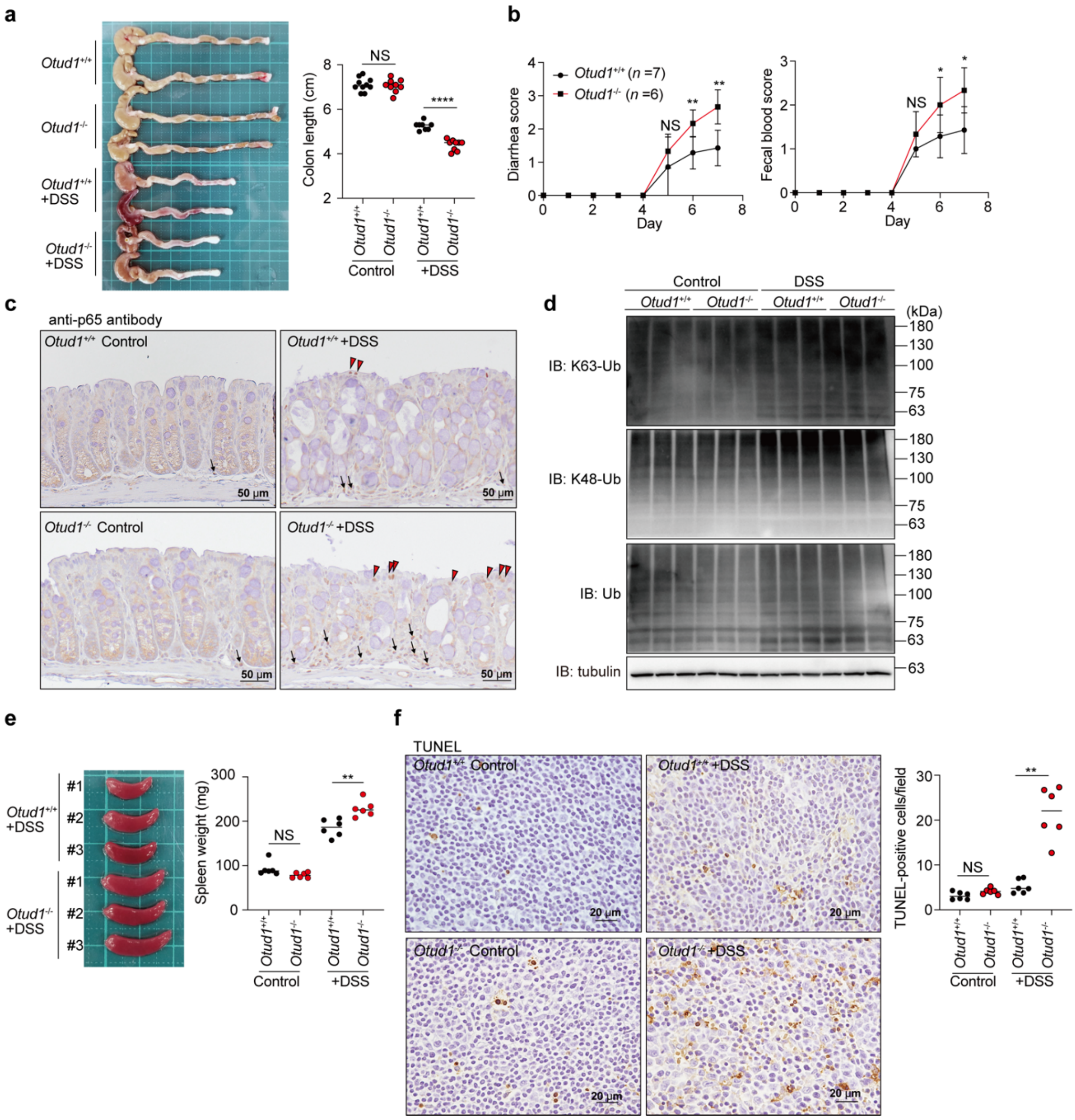

Supplementary Fig. 6 Increased susceptibility to DSS-induced colitis in *Otud1*$^{-/-}$-mice. a, Shortening of colon in DSS-administered *Otud1*$^{-/-}$-mice. Drinking water containing 2.5% DSS was administered to *Otud1*$^{+/+}$- and *Otud1*$^{-/-}$-mice for 7 days, and colon lengths were analyzed. Representative images (*left*) and lengths of colons (*right*, n = 8-10) are shown. **b**, Increased pathology scores in DSS-treated *Otud1*$^{-/-}$-mice. The scores for diarrhea and fecal blood in *Otud1*$^{+/+}$- (n = 7) and *Otud1*$^{-/-}$-mice (n = 6) were evaluated as described in the Methods. **c**, Increased intranuclear localization of p65 in colons from DSS-treated *Otud1*$^{-/-}$-mice. Specimens, prepared as described in Fig. 6a, were stained with an anti-p65 antibody. *Arrows*: inflammatory cells in lamina propria of colonic mucosa, *Arrowheads*: p65-positive colonic epithelial cells. *Bars*: 50 μm. **d**, Overall

ubiquitination was not affected in *Otud1*$^{+/+}$- and *Otud1*$^{-/-}$-mice with or without DSS-treatment. Colonic lysates from *Otud1*$^{+/+}$- and *Otud1*$^{-/-}$-mice treated with or without DSS were immunoblotted with a K63-linkage specific-, K48-linkage specific-, or pan-ubiquitin antibody as indicated. **e**, Splenomegaly in DSS-treated *Otud1*$^{-/-}$-mice. Images (*left*) and weights of spleens (*right*, *n* = 6) are shown. **f,** Increased cell death of splenocytes in DSS-treated *Otud1*$^{-/-}$-mice. TUNEL staining of spleen sections, and numbers of TUNEL-positive cells per 40x field (*n* = 6) were analyzed. *Bars*: 20 μm. **a**, **b**, **e**, **f**, Data are shown as scatter plots and were evaluated by the Mann-Whitney test. *: $P<0.05$, **: $P<0.01$, ****: $P<0.0001$, NS: not significant.

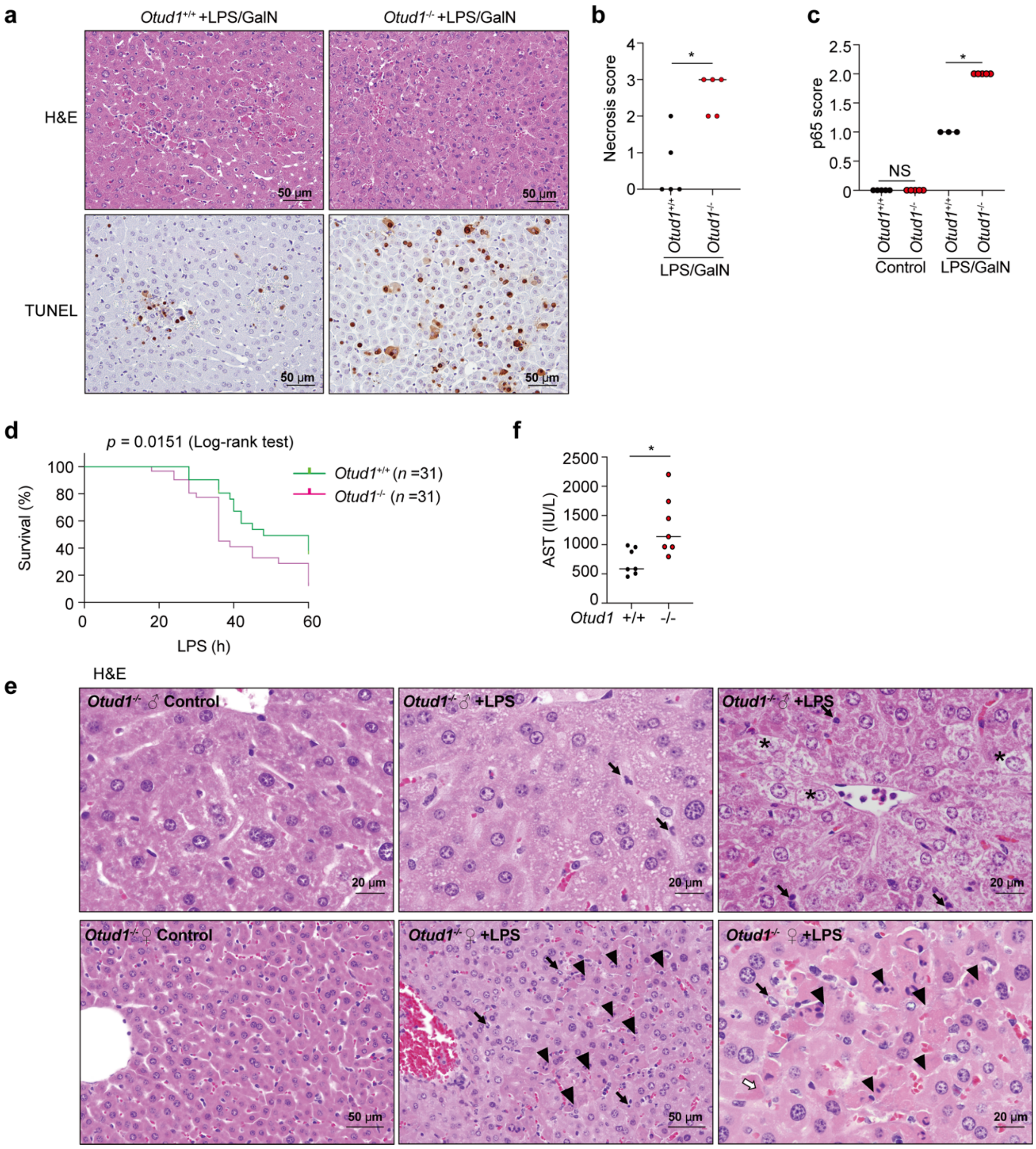

Supplementary Fig. 7 Exacerbation of LPS-induced liver damage in *Otud1*$^{-/-}$-mice. **a**, Enhanced cell death in livers from LPS/GalN-treated *Otud1*$^{-/-}$-mice. The higher magnification images of Fig. 6g are shown. *Bars*: 50 µm. **b**, Increased necrosis score in LPS/GalN-treated *Otud1*$^{-/-}$-mice. Hepatocellular death was scored on a semiquantitative scale of 0 to 3, as described in the Methods (n = 5). **c**, Increased intranuclear staining of p65 in hepatocytes in LPS/GalN-treated *Otud1*$^{-/-}$-mice. The nuclear staining of p65 was semi-quantitatively scored on a scale of 0 to 2, as described in the Methods. **d**, *Otud1*$^{-/-}$-mice are sensitive to LPS. Survival percentages of LPS (20 mg/kg i.p. injection) treated *Otud1*$^{+/+}$- and *Otud1*$^{-}$

$^{/-}$-mice (*n* = 31 each) determined by the Kaplan-Meier method are shown. Log-rank test was performed. **e**, Histopathological changes in the livers of *Otud1*$^{-/-}$-mice 24 h after LPS treatment. H&E stained livers from *Otud1*$^{-/-}$-male (*upper panels*) or *Otud1*$^{-/-}$-female mice (*lower panels*) with normal controls (*left panels*) or LPS-treated (*middle and right panels*) mice are shown. *Arrowheads*: necrotic hepatocytes; *arrows*: infiltration of inflammatory cells; *asterisks*: cell degradation. *Bars*: 20 or 50 µm. **f,** Increased AST level in LPS-treated *Otud1*$^{-/-}$-mice. AST levels in plasma from *Otud1*$^{+/+}$- (*n* = 7) and *Otud1*$^{-/-}$-mice (*n* = 7) after 36 h administration of LPS. **b**, **c**, **f**, Data are shown as scatter plots and were evaluated by the Mann-Whitney test. *: *P*<0.05, NS: not significant.

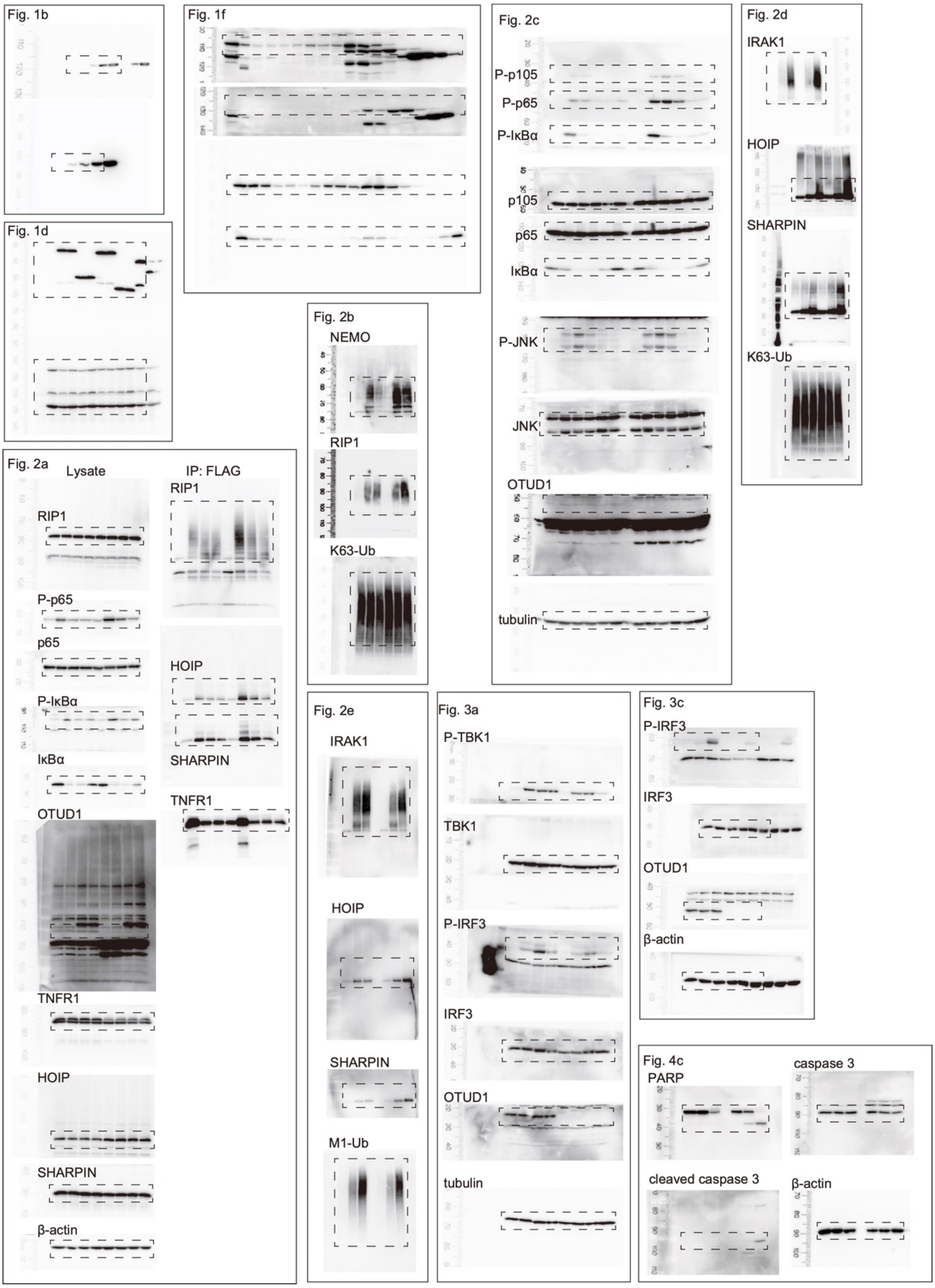

Supplementary Fig. 8 Uncropped immunoblotting images.

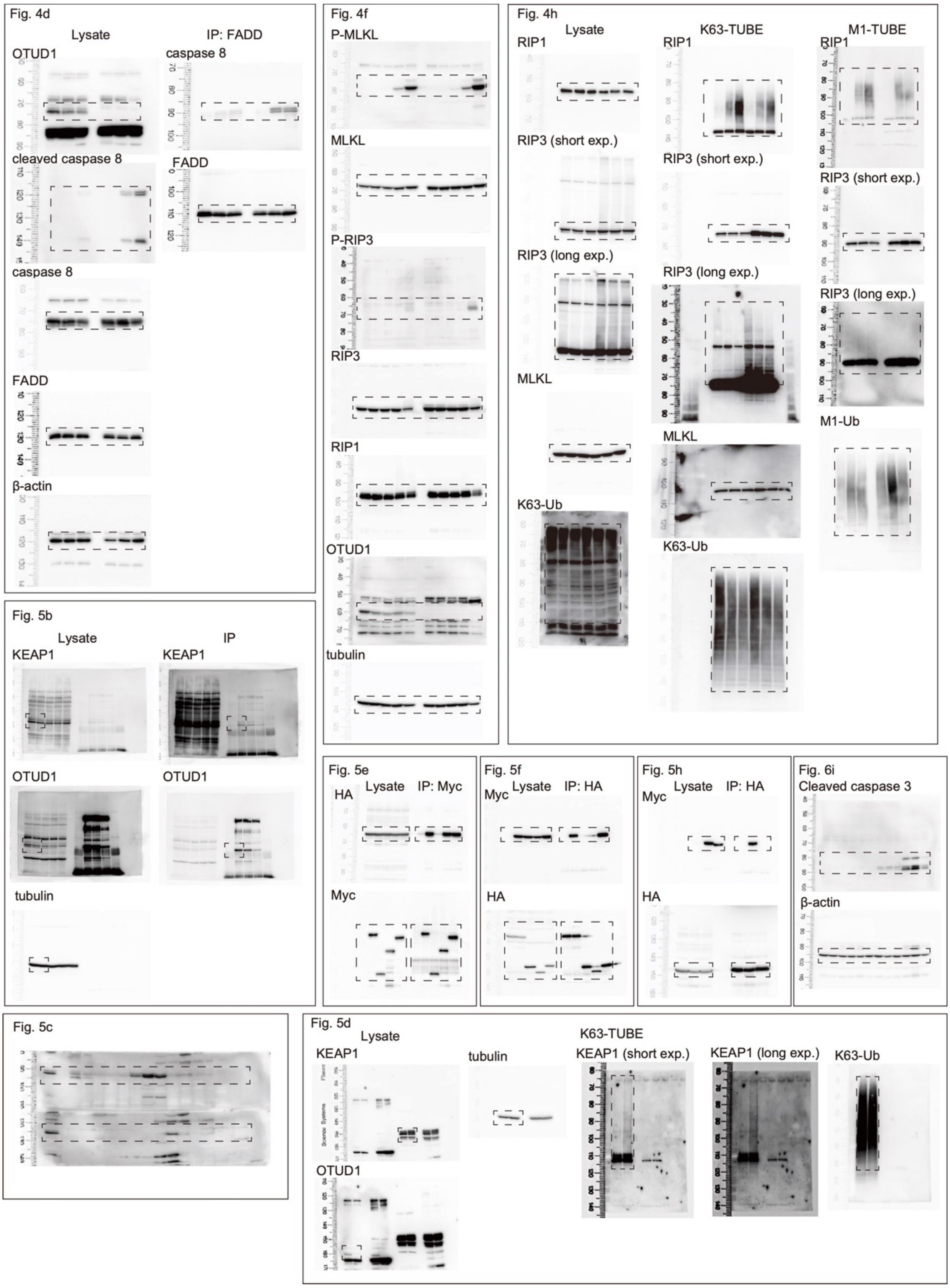

Supplementary Fig. 8 Uncropped immunoblotting images (cont.).
